# Coloured hearing, colour music, colour organs, and the search for perceptually meaningful correspondences between colour and sound

**DOI:** 10.1177/20416695221092802

**Published:** 2022-05-09

**Authors:** Charles Spence, Nicola Di Stefano

**Affiliations:** Crossmodal Research Laboratory, University of Oxford; Institute for Cognitive Sciences and Technologies, National Research Council of Italy (CNR)

**Keywords:** colour, pitch, sound, correspondence, synaesthesia, colour music

## Abstract

There has long been interest in the nature of the relationship(s) between hue and pitch or, in other words, between colour and musical/pure tones, stretching back at least as far as Newton, Goethe, Helmholtz, and beyond. In this narrative historical review, we take a closer look at the motivations that have lain behind the various assertions that have been made in the literature concerning the analogies, and possible perceptual similarities, between colour and sound. During the last century, a number of experimental psychologists have also investigated the nature of the correspondence between these two primary dimensions of perceptual experience. The multitude of different crossmodal mappings that have been put forward over the centuries are summarized, and a distinction drawn between physical/structural and psychological correspondences. The latter being further sub-divided into perceptual and affective categories. Interest in physical correspondences has typically been motivated by the structural similarities (analogous mappings) between the organization of perceptible dimensions of auditory and visual experience. Emphasis has been placed both on the similarity in terms of the number of basic categories into which pitch and colour can be arranged and also on the fact that both can be conceptualized as circular dimensions. A distinction is drawn between those commentators who have argued for a dimensional alignment of pitch and hue (based on a structural mapping), and those who appear to have been motivated by the existence of specific correspondences between particular pairs of auditory and visual stimuli instead (often, as we will see, based on the idiosyncratic correspondences that have been reported by synaesthetes). Ultimately, though, the emotional-mediation account would currently appear to provide the most parsimonious account for whatever affinity the majority of people experience between musical sounds and colour.

## 1. Introduction

For centuries now, commentators working in a wide variety of research fields have speculated on the possible (privileged) relationship that might exist between colour and sound (e.g., Goethe, 1840; Helmholtz, 1867; Newton, 1704). In fact, the intuition that there was a meaningful correlation between the musical scale and the rainbow spectrum of hues can be traced all the way back to ancient Greek philosophers such as Pythagoras (Moritz, 1997) and to Ptolemeus Alexandreus (Sebba, 1991). In his theorization concerning the senses, Aristotle also hypothesized the existence of a link between colour harmonies and musical proportions, speculating that the effect of colour combinations to the eye might depend upon the same numerical proportions as the musical sounds (Aristotle, 1908, III 439b–440a). In the contemporary era, such audiovisual crossmodal correspondences, as they are now more commonly known (see Spence, 2011), have started to fascinate a new generation of experimental psychologists (e.g., Adeli et al., 2014) and music theorists (e.g., Eitan, 2017). There has also been interest in colour-sound correspondences from those interested in the design of more intuitive sensory substitution systems (e.g., for those who are visually impaired; Hamilton-Fletcher et al., 2016; Marks, 1983).^1^ However, while some commentators have chosen to emphasize the similar structure of the perceptual spaces carved out by simple visual and auditory sensory experiences (Pridmore, 1992), others have instead focused their attention on the putative perceptual similarity between specific combinations of sensory inputs (e.g., see Kandinsky, 1977). The latter highlighted, for example, by the title of an article by Ione and Tyler (2004): ‘Is F-sharp coloured violet?’

The psychological affinity between simple auditory and visual stimuli can be further sub-divided into at least two distinct categories, namely perceptual and affective similarity (the latter, possibly linking to Kandinsky’s, 1977, notion, of ‘inner harmony’; see Spence & Di Stefano, 2022). While interest in the crossmodal correspondences between sound and colour initially appears to have been motivated by intuitions based around the structural and physical similarity (these so-called analogical mappings) between the two senses, the absence of any obvious phenomenological sense of perceptual similarity has led to the emergence of emotional mediation as a likely ‘glue’ linking (if not necessarily binding) the stimuli presented in this particular pair of sensory modalities. At the outset, though, it is important to consider the various perceptual dimensions underlying our experience of auditory and visual stimuli, as this has also proven to be a major motivator for empirical research, as well as a significant constraint on the associations that have been proposed to date. At the same time, however, and as we will come back to later in this narrative historical review, when considering the topic of ‘colour music’, it is important to recognize that as soon as auditory and visual stimuli are embedded within spatial and/or temporal patterns, as they typically are in works of art (and music), the likelihood is that intramodal perceptual grouping will completely override any weak crossmodal perceptual alignment that might otherwise have been detectable (see Sebba, 1991, on this point; and see Spence, 2015, for a review). In much the same way, the affective/mood associations that have been established between specific simple sensory stimuli when presented in isolation will likely also change as a result of any intramodal perceptual grouping that might occur. For instance, consider here only how the affective meaning of musical scales has been shown to depend on direction, i.e., whether they are ascending or descending (e.g., Collier & Hubbard, 2001).

### 1.1. Metathetic, Prothetic, and Circular Dimensions

More than half a century ago now, Stevens (1957) highlighted an important distinction between ‘metathetic’ and ‘prothetic’ stimulus dimensions. Loudness, brightness, lightness, heaviness, duration, roughness, area, and apparent length etc., are all prothetic dimensions, with a clear ‘more than’ and ‘less than’ end.^2^ Stevens refers to these as quantitative perceptual continua (i.e., having to do with how much) with ratio properties. According to Panek and Stevens (1966), the saturation of red can also be arranged as a prothetic continuum. Metathetic dimensions, by contrast, obey a well-structured organization without necessarily having a ‘more than’ or ‘less than’ end. In adults, prothetic dimensions tend to possess a unitary and well-ordered psychophysics, whereas metathetic stimulus dimensions do not. Stevens classified the latter perceptual continua as ‘what kind’ or ‘where (position)’. Relevant here, both auditory pitch and hue are metathetic dimensions, as are visual position, inclination, and proportion, according to Stevens. Smith and Sera (1992) subsequently added shape to the list. Interestingly, however, pitch and hue can also both be represented as circular dimensions. In fact, according to Pridmore (1992), they are the only perceptual dimensions that can be so represented (see Table 1 for a summary).

**Table 1.** Putative organizational principles underpinning various dimensions of perceptual experience, and supporting references.

Organization	Relevant examples	References
Prothethic ('How much')	Intensity; Magnitude; Loudness; Brightness; (Visual) lightness;^*^ Heaviness; Duration; Roughness; Area; Apparent length Saturation (of red)	Stevens (1957) Panek and Stevens (1966)
Metathetic ('What kind' or 'Where')	Laterality; Pitch; Hue category; Position Shape	Stevens (1957) Smith and Sera (1992)
Polar	Elevation (Up-down); Size (big-small);	Clark (1970); cf. Gardner (1974); Proctor and Cho (2006)
	Bitter-sweet	Crisinel et al. (2012); though see Watson and Gunter (2017)
	Various semantic differential scales	e.g., Wicker (1968); Oyama et al. (1998); Pedović and Stosić (2018)
Circular	Hue; Pitch	Pridmore (1992)
Amodal (Universal)	Sensory brightness; Intensity; Rate; Duration; Spatial location; Spatial extent; Rhythm; Shape Size; Texture; Flexibility; Duration; Intensity	Von Hornbostel (1931, 1950); Lewkowicz and Turkewitz (1980); cf. Smith (1987) Walker-Andrews (1994)
No obvious organizational principle	The class of all basic tastes (sweet, sour, bitter, salty, umami); The class of all odorants/flavours (e.g., creamy, meaty, floral, citrus, herbal)	

* According to Marks (1987), while visual lightness is a prothetic dimension, it is extremely unusual in as much as which end of the dimension is associated with 'more', and which with 'less’, varies between individuals. Note that a given dimension of perceptual experience (e.g., brightness or pitch) can appear in more than one organizational category.

Polar dimensions should also be considered here. According to Clark (1970), the latter are fundamental to children’s development of relational concepts. Dimensions such as big vs. little, high vs. low, but presumably also left vs. right, sweet vs. sour/bitter are all polar. Notice here how a polar organization can be applied to prothetic dimensions (big vs. little; spatial high vs. low), to metathetic dimensions (such as left vs. right; high vs. low pitch), and to pairs of stimuli that cannot be organized as part of either prothetic or metathetic dimensions, such as between pairs of basic tastes (though see Crisinel et al., 2012; and Watson & Gunter, 2017) or olfactory stimuli. Polar dimensions can presumably be aligned with other polar dimensions although which poles people choose to align may well sometimes turn out to be arbitrary. Intriguingly, certain of the basic tastes have recently been shown to correspond to the polar dimension of elevation (e.g., high vs. low; see Velasco et al., 2019).

While it is undoubtedly possible to establish an analogy between pitch and hue at more of a cognitive, or abstract, level, and by so doing relate, or figure out, the relative position of auditory and visual stimuli along their respective unisensory dimensions (cf. Cohen, 1934; Mellers & Birnbaum, 1982; Moul, 1930; Simpson et al., 1956, p. 100),^3^ there is little empirical evidence supporting the existence of any kind of perceptual ‘resonance’ (cf. Muecke & Zach, 2007), or unification^4^ that necessarily follows from the correct structural alignment of stimulus dimensions (e.g., Sebba, 1991). That is, the dimensional alignment of pitch and hue does not necessarily lead to any perceptually apparent correspondence based on the perceived similarity of the component stimuli.^5^

Intriguingly, von Hornbostel (1931, 1950) was convinced that sensory brightness represented one of the universal dimensions of sensory experience. His participants were required to match sounds of different pitches to points along a greyscale. He also had them crossmodally match scents with grayscale values. He took his results, and, in particular, the transitivity between different crossmodal comparisons that they apparently revealed, to demonstrate that the concept or quality of ‘sensory brightness’ was one that was common to all of the senses. Were this to be the case, one could easily imagine how simply matching the brightness of auditory and visual stimuli would provide a means of objectively aligning the senses. However, subsequent researchers were not convinced (see Cohen, 1934), arguing instead for a relative/relational judgment rather than necessarily a crossmodal perceptual mapping (see also Hartshorne, 1934). As Cohen (1934, p. 119) put it, the stimuli were ‘analogous’ rather than ‘identical’. Cohen explains as follows: “It would not be unreasonable then to suppose that cross-modality comparison should be based (physiologically, if not introspectively) upon relative positions within different ‘absolute’ scales. According to this view equation with respect to brightness of two experiences of different modalities would involve nothing more than the identity of relative positions upon two wholly independent scales.”

Echoing von Hornbostel’s theoretical speculation, Smith (1987, p. 96) writes that: “we have a lot to gain if we can get beyond mapping specific dimensions one to another and instead delineate the amodal dimensions.” The possibility for amodal concept(s) to exist is apparently linked to the existence of absolute correspondences, as Smith (1987, pp. 97–98) observed: “This suggestion of a trend from dichotomous, categorical treatments of continua to more relativistic ones ought not to be confused with the issue of absolute versus relative correspondences across dimensions. The notion of absolute correspondences between dimensions is that particular values on one dimension map onto particular values on another—for example, higher is not *like* brighter; rather, a specific pitch matches a specific brightness. As Marks et al. point out, there is little evidence for such absolute correspondences.” (Italics in original). Interestingly, Mellers and Birnbaum (1982, p. 600) came to much the same conclusion, namely that: “In cross-modality judgments, the scale values are influenced by the stimulus distribution: It appears that subjects compare the relative position of a stimulus in its distribution with the relative position of a stimulus of another modality to its distribution, going on to suggest that their results were consistent with a psychological relativity theory of cross-modality judgment.”

To summarize, beyond shedding light on the way in which stimuli are organized within perceptual dimensions in different sensory modalities, the distinction between metathetic and prothetic stimulus dimensions leads to the related distinction between absolute vs. relative crossmodal correspondences. The latter distinction is, in turn, instrumental when it comes to assuming the existence of an amodal concept, conceived of as the same physical property (such as shape) being picked up via multiple senses (see Lewkowicz & Turkewitz, 1980). This, it should be noted, is subtly different from von Hornbostel’s (1931, 1950) notion of universal dimensions of perceptual experience. The emphasis in the latter case would appear to be on the perceptual experience itself (i.e., what it is like), whereas the emphasis for those who have proposed amodal dimensions is on the multiple routes to picking-up information about physical properties out there, regardless of the perceptual qualities that may be associated with that information.

### 1.2. Sensory Scaling

Researchers have long known that people can scale sensations in different sensory modalities across a wide range of prothetic stimulus dimensions (Stevens, 1966; see also Bond & Stevens, 1969). And while some have wanted to argue from such matching behaviours that there may be shared attributes of experience, such as sensory brightness in von Hornbostel’s (1931) case, it turns out that it is hard to rule out alternative, more cognitive, accounts of the underlying process, as we have just seen (see Cohen, 1934). That said, Marks et al. (1987, p. 84) conclude that: “In some fundamental sense, the similarities between pitch and brightness and between loudness and brightness are personal, internal, and subjective; they reside in perception per se and probably depend on common processes of neural coding.” Note the strong claim here, albeit with multiple provisos, that similarity relations are perceptual in nature. Nevertheless, the ‘personal, internal, and subjective’ element did not stop Marks (1974a, b) from trying to establish a robust psychophysics based on the crossmodal matching of the colour lightness of grey surfaces with the pitch of pure tones.^6^ However, the ability to crossmodally match stimuli is presumably possible between any pair of prothetic stimulus dimensions, only a few of which might be argued to pick-up on the same stimulus, or perceptual, property (cf. Cohen, 1934).^7^ Elsewhere, Marks et al. (1987, p. 5) talk of the “perceptual, cross-modal equivalence with respect to intensity”. As such, it may be difficult to assert anything concrete about the perceptual similarity of pairs of stimuli based merely on the fact that participants can engage in robust crossmodal matching of stimuli relative to the dimensions to which they belong. In contrast to Marks and colleagues’ suggestion that the similarities are fundamentally perceptual in nature, we will argue for an emotional mediation account of colour-sound correspondences instead (cf. Wilms & Oberfeld, 2018).

Here it may also be worthwhile to contrast hue and pitch with other dimensions of unisensory experience, namely basic tastes and odour qualities which can neither be organized prothetically nor metathetically. Notice how the latter can both be distinguished from one another (i.e., sweet can be distinguished from salty and sour etc.) without there necessarily being any obvious organizational structure linking the various stimuli within each category (i.e., tastes and odours, respectively).^8^ The organization of smell and taste qualities are, in other words, neither prothetic nor metathetic. Nevertheless, research on the crossmodal correspondences now clearly reveals the existence of robust associations between colour hue categories (such as red, white, etc.) and basic taste qualities, such as sweet, bitter, salty, sour, salty, and umami (Spence & Levitan, 2021; Spence et al., 2015; see also Ikeda, 2002), as well as with odour qualities (see Spence, 2020b, for a review).^9^ Crossmodal correspondences have also been documented between auditory pitch and both basic taste qualities (e.g., Knöferle et al., 2015), and various olfactory stimuli (e.g., Belkin et al., 1997; Crisinel & Spence, 2012; see Di Stefano et al., 2022; Spence, 2021, for reviews).

Given the latter observation, it would appear that the nature, or structure, of the underlying stimulus/perceptual dimension (see Table 1) does not necessarily constrain the likelihood of observing, or establishing, crossmodal correspondences, nor does it automatically connect to the notion of perceptual similarity. Taken together, therefore, the research that has been reviewed in this section would appear to support the claim that the strength and quality of crossmodal correspondences, such as, for example, between pitch and hue, does not necessarily bear any meaningful relation to the structure, or organization, of the underlying perceptual continua. This conclusion obviously runs counter to the intuitions of those who first commented on the connection between colours and musical sounds.^10^ Indeed, it should be noted that the exact correspondence between these two classes of continua and their underlying neural representations is by no means clear (Lewkowicz & Turkewitz, 1980; Stevens, 1971).

It is worth noting how the conclusion that stimulus dimensional structure has no implications for crossmodal perceptual similarity would appear to contradict the claims made by the psychologist Lawrence Marks in his influential book, *The unity of the senses*, when he asserts that: “Sensory correspondence is not a domain of inquiry restricted to scientists, a matter solely for experimental scrutiny and empirically based theory. The plain fact is that sensory analogies do exist; they are important to the ways that we sense, perceive, and cognize; they are significant properties of the bodies and minds of people” (Marks, 1978, p. 7). In contrast, according to the view espoused here, while ‘sensory analogies’ may bias (or be based on) the kinds of sensory connections that people find it easy to establish cognitively, we do not believe that they play any role in determining the nature, or strength, of the crossmodal correspondences that are based on perceived similarity. However, while apparently contradicting Marks’ view, our own conclusion would appear to be much more in tune with the art historian Gombrich’s suggestion that we should focus our attention on the structural relationships in the system rather than on the similarity of the elements: “The problem of synesthetic equivalences will cease to look embarrassingly arbitrary and subjective if . . . we fix our attention not on likeness of elements but on structural relationships within a scale or matrix” (Gombrich, 1960, p. 314).

### 1.3. On the Multiplicity/Hierarchy of Crossmodal Correspondences

One other important point to bear in mind here is that, outside of the psychophysics laboratory, perceptual stimuli typically vary along several dimensions simultaneously (e.g., two visual stimuli may well differ in terms of their hue, but also in terms of their size, shape, texture, etc.). There is presumably a hierarchy of crossmodal correspondences, such that certain perceptual dimensions (or attributes) may only obviously be aligned if the more natural, or perceptually salient, matching dimensions (or attributes) are not available (cf. Gardner, 1974; Parise, 2016). That is, the participants in laboratory studies will presumably simply go with the best of the available response options when, say, in an experimental setting, they are instructed to match a colour to a musical sound. In the audiovisual context, note only how auditory pitch, a correlate to visual brightness, is overall at least as strong as the crossmodal correspondence with loudness. In fact, according to Marks (1989, p. 598), it may even be stronger, or more dominant. In other words, one can ask both what is the spontaneously chosen dimension corresponding to a given perceptual experience, and whether it is consensually chosen across individuals. Separately, for a given specific pairing of attributes or dimensions one can ask how consistent it is across individuals (see Spence, submitted). It is interesting to note how the former question has rarely been posed by researchers, with the latter normally assuming what the comparison dimension should be.

As such, merely demonstrating a statistically significant crossmodal correspondence between stimuli in two arbitrary perceptual dimensions (such as pitch and hue) does not necessarily mean that the correspondence so documented reflects the best of all possible matches with either of the component attributes, merely that it is the best of the options that happened to be available to participants at the time they were asked (see Spence & Levitan, 2021; Spence et al., 2015, on this point). Thus, the mere fact that a robust crossmodal correspondence can be established between two stimuli, or stimulus dimensions, does not mean that this will necessarily be the most natural, or obvious, association that an observer will make, or be drawn to, spontaneously should there be a better-connected dimension available (cf. O’Mahony, 1983, on the multiple correspondences with basic tastes, of which hue is but one). Notice here also how the frequency, or consensuality, with which specific crossmodal associations occur is not necessarily linked to the strength, or vividness, of the association itself (e.g., Marks, 1975; Rader & Tellegen, 1987; Spence, submitted). Or, to give another example, consider only how people may draw an analogy between spatial patterns in vision and temporal patterns in audition if those happen to be the only dimensions in which the auditory and visual stimuli differ/vary (Julesz & Hirsh, 1972). However, as soon as a temporal pattern is presented visually then this may come to dominate as the more natural, or intuitive, crossmodal match for an auditory temporal pattern than a spatial pattern.

### 1.4. On the Popularity of Colour-Sound Correspondences

So why, then, is it that people have been so interested in colour-pitch correspondences for so long? The topic is undoubtedly of theoretical interest in terms of the discussion of crossmodal perceptual similarity, with early interest seemingly stemming from the various structural and physical similarities that have been highlighted between the dimensions of stimuli/experience, though as we will see later (see **Section 4**), interest was undoubtedly revived as a result of parallel developments in the emerging artistic field of colour-music (e.g., Klein [Cornwall-Clyne], 1937; see Zilczer, 1987, p. 2016, for reviews). It is, though, worth stressing how many of the artists working in the field of colour-music (not to mention many of the prominent research scientists working in the area) have, over the years, been distracted by the search for crossmodal mappings between musical features and colour (and/or form) in the idiosyncratic experiences reported by synaesthetes (see Donnell-Kotrozo, 1978; Galeyev, 1976, 2003; Itoh et al., 2017; Kandinsky, 1977; von Erhardt-Siebold, 1932; Zilczer, 1987).^11^ Indeed, the florid concurrents that have so often been experienced, and reported, by coloured-hearing synaesthetes have undoubtedly helped to raise awareness/interest in crossmodal mappings between this particular pair of sensory attributes (Hänggi et al., 2008; Menouti et al., 2015). In recent decades, the growing interest in sensory substitution systems/devices (e.g., for the blind) has also drawn attention to the question of how best to ‘translate’ visual attributes, such as colour, into sound in an intuitively meaningful manner (see Hamilton-Fletcher et al., 2016; Marks, 1983; see also Lupton, 2018).

### 1.5. Coloured Hearing Synaesthesia

While some commentators have wanted to align the visible colour spectrum with the auditory pitch scale (Newton, 1704; see also Arnheim, 1986), others have focused instead on specific matches between particular pairs of stimuli, often based on the idiosyncratic inducer-concurrent relations experienced by those synaesthetes who report experiencing coloured music (see Suarez de Mendoza, 1890). For example, just take the Bauhaus/Abstract artist Kandinsky’s (1977) suggestion that the sound of the trumpet is scarlet (see Ione & Tyler, 2003, 2004; Just, 2017; though see also O’Regan, 2011). At one point, Kandinsky (1977, p. 40) writes that: “Light warm red …In music, it is a sound of trumpets, strong, harsh, and ringing.” In this particular case, one can only wonder whether it is mere coincidence that a couple of centuries earlier both Locke (1690) and Leibniz (1704/1896) had written about the blind man who apparently understands what scarlet is because of its being compared to the sound of the trumpet. Such consistency might be taken to suggest the existence of a meaningful (i.e., fundamental) mapping between a specific timbre and a particular hue category (see also Lavignac, 1899; Wallmark, 2019). Other commentators, though, have wanted to put forward an account in terms of learned associations instead. Just take, for example, the eminent neurologist MacDonald Critchley (1900-1997), who once apparently suggested that “the familiar story of trumpet blasts provoking a photism of red, may stem from the fact that such a sound immediately calls up in some persons an imagery of soldiers on parade. Ordinarily they shall be in dress uniform. This evokes a mental picture of scarlet. Should the middle part of this notion eventually become submerged, there will remain a synaesthetic linkage of trumpet-calls with redness.” (as quoted in Harrison, 2001, p. 209; cf. Barilari et al., 2018, for the suggestion that crossmodal correspondences involving visual stimuli may be internalized by the early blind in terms of the structural regularities in language). It is perhaps also worth considering the effect of key colour on the recognition of absolute pitch on the piano (Marvin & Brinkman, 2000).

Kandinsky (1977) referred to a number of specific colour-sound mappings in his writing, though it is often unclear whether the examples he gives constitute examples of the artist’s own synaesthesia, or should be better considered as examples of emotionally-mediated crossmodal correspondences (and hence might perhaps be expected to be experienced by us all; Spence, 2020a). Kandinsky often refers to the latter in terms of ‘inner harmony’ (cf. Harrison, 2001). Something of a similar challenge has faced those interested in trying to understand more about the idiosyncratic crossmodal mappings that have been suggested by synaesthetic Russian artists, namely the composers Rimsky-Korsakov (who reported ‘seeing’ music in the key of A-major as yellow; Myers, 1911), and Scriabin (Galeyev & Vanechkina, 2001; Myers, 1914). Once again, though, it has long been the subject of debate as to what exactly the relationship, if any, was between Scriabin’s personal repertoire of idiosyncratic audiovisual inducer-concurrent mappings, and those that he chose to incorporate into his colour circle/score/luce (Galeyev & Vanechkina, 2001; Triarhou, 2016).^12^

‘Coloured hearing’ turns out to be one of the most commonly-mentioned forms of synaesthesia, and often appeared in the scientific literature in the decades either side of 1900 (e.g., Dauriac, 1902; de Parville, 1883; English, 1923; Flournoy, 1893; Ginsberg, 1923; Jewanski et al., 2009; Jewanski et al., 2011; Jewanski, Simner, Day, Rothen, & Ward, 2020; Underwood, 1893).^13^ Marks (1975) provides a particularly thorough review of the multitude of early studies of coloured music and coloured speech sounds.^14^ Interestingly, both pitch and timbre appear to be salient features (i.e., sensory inducers) driving the coloured musical concurrents that have been reported to date. At the same time, however, it has also been acknowledged that there may be a strong visual mental imagery component to many coloured responses to music (Karwoski et al., 1942; Mudge, 1920; see also Spence & Deroy, 2013). And while it has been suggested that the inducer-concurrent mappings experienced by synaesthetes do typically tend to be appreciated by non-synaesthetes (e.g., see Ward et al., 2008), that presumably has no necessary implications regarding the question of whether the inducer is perceptually (or necessarily even affectively) similar to the concurrent in the case of synaesthesia.

## 2. Early Suggestions Concerning the Alignment of Colour and Pitch

**Figure 1.**
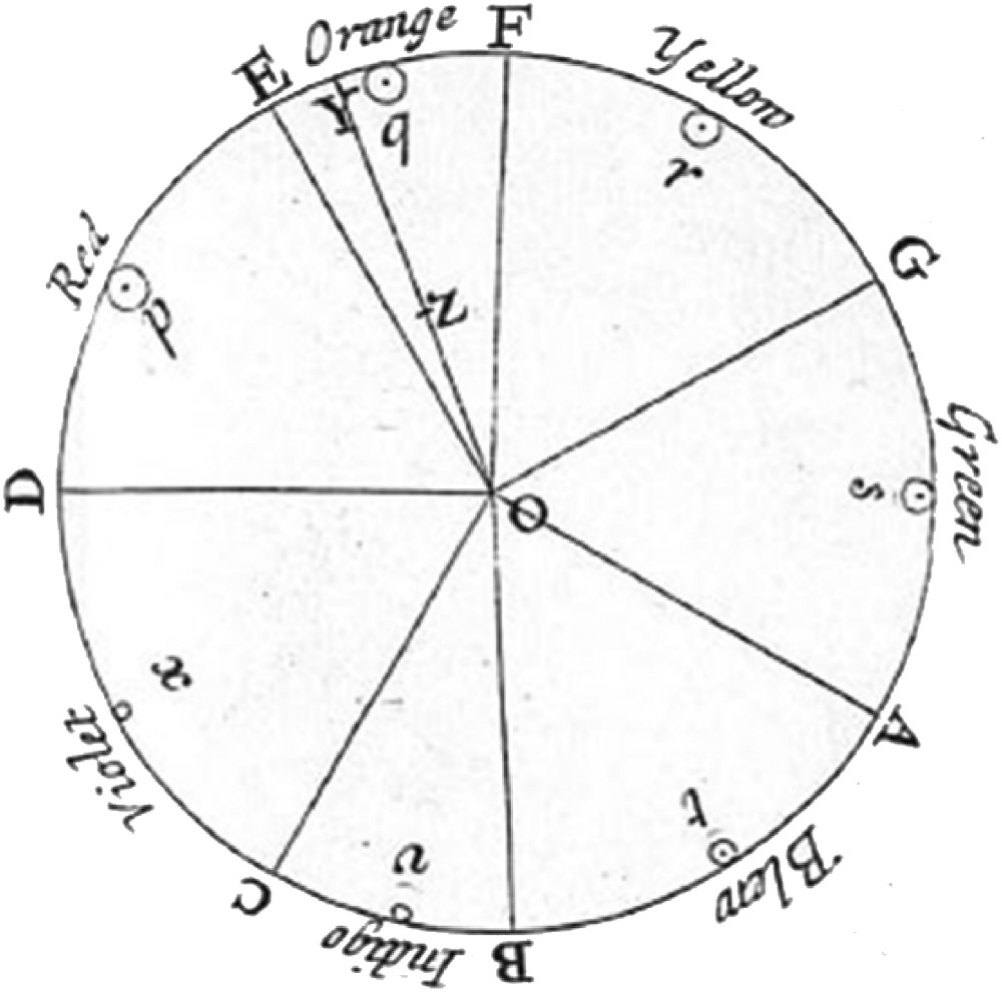
Newton’s suggested analogy between the seven musical notes and the seven spectral hues; The musical divisions of the prism, as proposed by Newton (1704). The seven colours: red, orange, yellow, green, blue, indigo and violet, filled the seven intervals between the eight notes, starting from the highest note on the right. Deep violet, on the left, is the most refracted light, and red on the right is the least refracted.

As has been noted already, of all the crossmodal correspondences that could potentially have captured the interest of commentators (see Spence, 2011, for a partial listing), it is the connection between colour and pitch that would seem to have attracted by far the most widespread, and longstanding, interest amongst everyone from philosophers to writers, and from scientists to artists. But, once again, we return to the question of why that should be? Is this particular pairing of sensory modalities (or rather stimulus dimensions) in some way special? Otherwise, how are we to explain what has made it stand out from the many other crossmodal correspondences that we now know about? As we have just seen, the answer would not appear to reside in the structural similarity between these stimulus dimensions. Interest from the late nineteenth century is tied up with the colour music so often reported amongst artists/in the press (Zilczer, 1987). However, looking even further back in time, one finds Sir Isaac Newton (Newton, 1704, book III, part I, qu. 13-14), famously drawing an analogy between the seven notes of the diatonic scale and the seven putative primary colours of the spectrum (Jewanski, 2010; see Figure 1).

**Figure 2.**
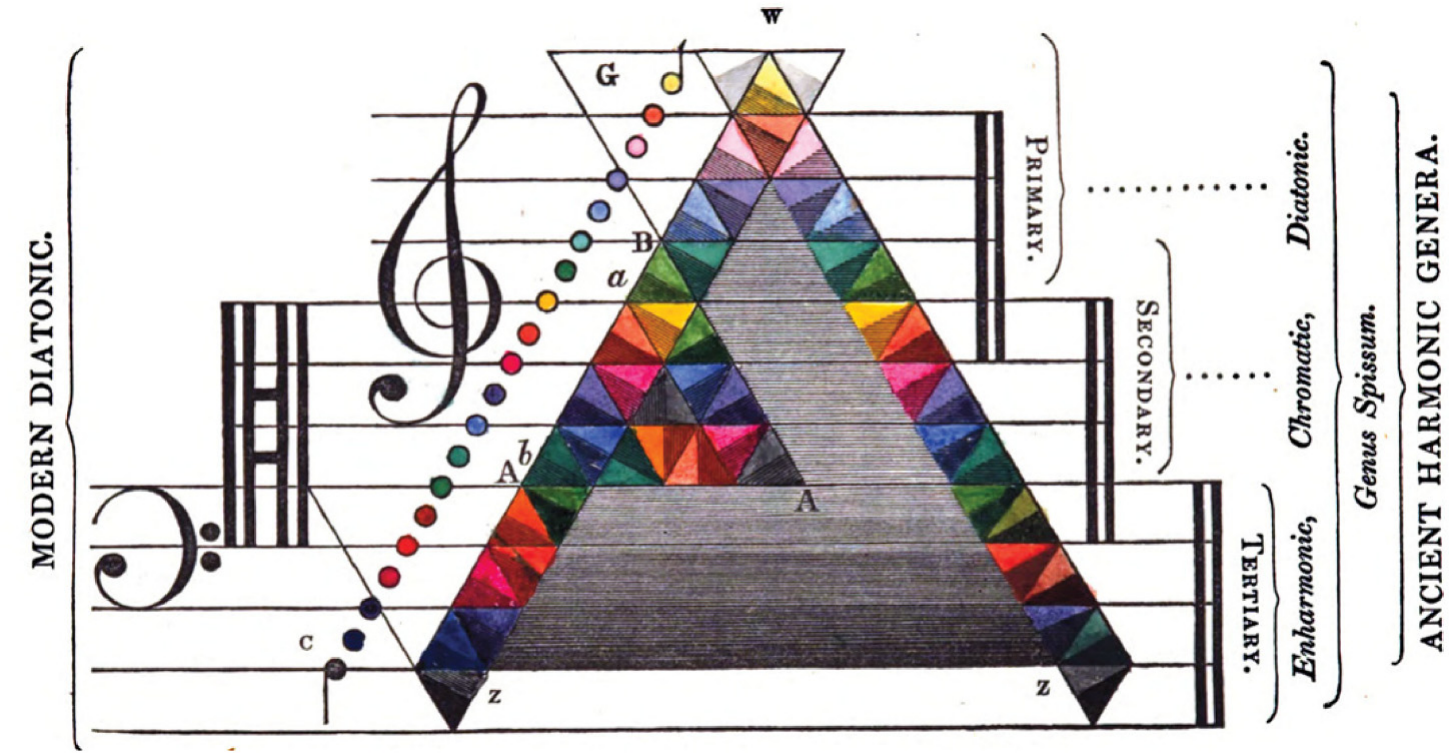
The correspondence between colours and musical sounds as theorized by Field (1835, p. 79). Each coloured triangle is divided into two equal triangles of slightly different hues that correspond to the chromatic intervals (represented also in circles on the left). Pitches are ordered from low to high, with darker and lighter hues, respectively.

Inspired by Newton’s *Opticks,* first published in 1704, the English chemist Field (1835) conceived colour painting based on musical criteria, leading to the definition of the general mapping between sound and colour. The systematic use of musical notions for describing the harmony of colours likely makes Field’s system the most rigorous and musically informed attempt to achieve a general theory of harmony which applies equally to music and sounds (see Figure 2; see also Spence & Di Stefano, 2022). Johannes Wolfgang von Goethe (Goethe, 1840, c. 201-202, para. 748) once also famously suggested an association between colour and musical key expressed as a general tendency to match darker hues to musical keys with flats in their signatures, and brighter colours to those with sharps. Or, as Goethe himself put it: “It would not be unreasonable to compare a painting of powerful effect with a piece of music in a sharp key; a painting of a soft effect with a piece of music in a flat key” (Goethe, 1840, p. 342).”^15^ Goethe also wrote: “That a certain relation exists between the two, has been always felt; . . . Colour and sound do not admit of being directly compared together in any way, but both are referable to a higher formula, both are derivable, although each for itself, from this higher law” (Goethe, 1840, p. 298). Notice how the stress here would appear to be on a structural analogy, or affective similarity, rather than on a direct crossmodal mapping driven by any perceived similarity between the component stimuli.

Subsequently, Hermann Ludwig Ferdinand von Helmholtz (1867, p. 237) described the following analogies between the notes of the piano and the colours of the spectrum: G, Red; G#, Red; A, Red; A#, Orange-red; B, Orange; c, Yellow; c#, Green; d, Greenish-blue; d#, Cyanogen-blue; e, Indigo-blue; f, Violet; g, g#, a, a# Ultra-violet; b, end of the solar spectrum. According to Kubovy and Van Valkenburg (2001), this particular scale extends to about a Fourth beyond the Octave.^16^ However, in the same multi-volume work, one also finds the famous German psychophysicist arguing that: “The distinctions among sensations which belong to different modalities, such as the differences among blue, warm, sweet, and high-pitched, are so fundamental as to exclude any possible transition from one modality to another and any relationship of greater or less similarity. For example, one cannot ask whether sweet is more like red or more like blue. Comparisons are possible only within each modality; we can cross over from blue through violet and carmine to scarlet, for example, and we can say that yellow is more like orange than like blue!” (Helmholtz, 1878/1971, p. 77).^17^ While the two claims made by Helmholtz might, at first, appear to be contradictory, it is presumably entirely possible to recognize an analogy between two physical stimulus dimensions without presupposing that the corresponding stimuli on the respective unisensory scales will necessarily be perceived as any more (or less) perceptually similar than other pairs of mismatching stimuli.

However, it is important to note that not everyone has necessarily agreed with Helmholtz’s bleak assessment concerning the possibility of experiencing perceptual similarity between the senses. Just take the following quote from Lawrence Marks, writing in 2011 about “perceptual similarities between and among sensory experiences in different modalities. Much as the colour aqua is more similar phenomenologically to cerulean than to pink, the flavour of lime more similar to lemon than to banana, so too are low notes played on a bassoon or an organ more like dark colours such as brown or black than bright colours such as yellow or white, while the higher notes played on clavier or a flute resemble yellow or white more than brown or black.” (Marks, 2011, p. 52). Unfortunately, though, Marks makes no reference to Helmholtz’s prior work, and hence it is not possible for us to know how he would respond to what appear to be the latter’s diametrically-opposed position.

Where Newton, Goethe, and Helmholtz led in terms of laying out their analogical mappings between musical notes and spectral colours, various other artists, musicians, composers, and inventors have all come out with their own slightly different correspondence tables mapping colours to the pitch of musical notes (see Table 2 for a number of the proposed correlations/associations between colour and chromatic musical scales). And while early interest was seemingly driven more by theoretical considerations related to the existence of a natural structural alignment (or analogy; see Jewanski, 2010) between these stimulus dimensions of perceptual experience, the subsequent development of such crossmodal tables has often been motivated more by the practical interest in trying to create colour organs and/or ‘light symphonies’. The latter inspired/facilitated by technological developments and ideas circulating in the world of abstract art (Zilczer, 1987).

**Table 2.** Correlation of colour and chromatic music scales. Table highlighting the various crossmodal correspondences that have been proposed since Newton. Newton’s correlation conforms to the seven-tone scale which he was probably familiar with. Castel’s correlations were made with the 12-tone chromatic scale, but as Wells (1980) notes, the hues fall in frequency as the tones rise in frequency. The alignment is reversed for the following correlations. (The scale, attributed to E. G. Lind, presents the pitch of tones (sound frequency, Hz) and the frequency of light (presented in parentheses in the Table as 10-8 Hz, for example red is 476 × 108 Hz).) [Reprinted with permission from Wells (1980, Table 1).].

Note	Newton	Castel	Finn	Lind	Maryon
	1700	1720–1735	1881	1900	c. 1920
C	Red	Blue	Red	259 Hz, red (476)	Red
C#		Sea green, blue-green	Vermillion		Red-orange
D	Orange	Green, bright green	Orange	289 Hz, orange (511)	Orange
D#		Olive, yellow-green	Yellow		Orange-yellow
E	Yellow	Yellow	Yellow-green	322 Hz, yellow (546)	Yellow
F	Green	Apricot, yellow-	Green	342 Hz, green (588)	Yellow-green
		orange, aurora			
F#		Orange	Blue-green		Green
G	Blue	Red	Turquiose blue	385 Hz, blue (630)	Blue-green
G#		Crimson	Blue		Blue
A	Indigo	Violet	Indigo	427 Hz, indigo (665)	Blue-violet
A#		Agate, blue-violet,	Violet		Violet
		light purple			
B	Violet	Indigo	Purple	485 Hz, violet (721)	Violet-red

## 3. Experimental Studies of Crossmodal Correspondences Between Colour and Brightness, Pitch, and Timbre

### 3.1. Colour-Pitch Correspondences

During the middle decades of the twentieth century, a number of experimental psychologists, often without any reference to the earlier studies (such as those mentioned above), set themselves the task of trying to establish the nature (and consequences) of any crossmodal correspondences that might exist between colour and pitch. According to Kubovy and Van Valkenburg (2001, p. 114), the justification for this line of empirical research was simply that: “early psychologists concluded that colour gets mapped onto pitch, since they are non-spatial and non-temporal, and since they are both caused by waves.” Notice here how the justification is in terms of the physical nature of the stimuli themselves.

In terms of empirical research, one of the first studies to assess colour-tone associations systematically in a non-synaesthetic population was reported by Simpson et al. (1956). These researchers investigated the nature of any crossmodal associations between colours and pure tones in 995 elementary school children (between third and sixth grade). The children were presented with each of six pure tones (125, 250, 1,000, 4,000, 8,000, and 12,000 Hz, or cycles per second, c.p.s.)^18^ at 40 and 50 dB and forced to choose which of six spectral colours (violet, blue, green, yellow, orange, and red) they ‘thought of’ immediately upon hearing each of the tones. Yellow and green were predominantly associated with high pitch. They suggested red and orange could be categorized as ‘middle-pitched’ colours, while violet and blue were predominantly ‘low-pitched’ colours.^19^ Here, though, it is worth mentioning the point made earlier concerning the fact that the participants were only ever able to choose the best of the six available options. Hence, were it to be the case that pink were to have been the optimal match to one of the pure tones, say, then this would not be apparent from the data.

Simpson et al.’s (1956) study was phrased as a game with the children themselves told to guess if they were unsure. It is therefore unclear whether the mappings that were captured were cognitive in origin or anything more (i.e., perceptual crossmodal correspondences). And while Simpson and colleagues claimed to have demonstrated crossmodal pitch-hue correspondences (or ‘synaesthesia’ in their misleading terminology),^20^ several researchers have subsequently questioned whether these researchers actually controlled the brightness and/or saturation of their stimuli (cf. Wicker, 1968). In the absence of certainty on this latter point, the possibility must remain that the crossmodal correspondences reported in this early study might reflect nothing more than a pitch-brightness correspondence (see Bleuler & Lehmann, 1881; Flournoy, 1893; Marks, 1982; and Riggs & Karwoski, 1934) rather than necessarily having anything to do with hue categories *per se* (Spence, 2011). Certainly, when other experimenters have subsequently looked for correspondences between hue and pitch, it is striking how many of them have failed in their efforts (e.g., Bernstein et al., 1971; Wicker, 1968) (see Table 3 for a summary).

**Table 3.** Summary of documented crossmodal correspondences between a selection of perceptual qualities of sounds and visual colour (and shape). Null results preceded by a '-ve:'.

Visual attribute		Auditory attribute		
	Pitch	Timbre	Loudness	'Complexity'
*Hue category*	Simpson et al. (1956);* Hamilton-Fletcher et al. (2017); Sun et al. (2018); Colour words: Marks et al. (1987); '-ve: Bernstein et al. (1971); '-ve: Wicker (1968)	Mudge (1920) Adeli et al. (2014) Reuter et al. (2018)	Hamilton-Fletcher et al. (2017)	Scriabin (see Galeyev & Vanechkina, 2001)
*Lightness/brightness/luminosity*	Mudge (1920); Root & Ross, 1965; Wicker (1968); Marks (1987); Melara (1989); Hubbard (1996); Sun et al. (2018); Anikin and Johansson (2019)		cf. Woodworth and Schlosberg (1954, p. 364) Stevens and Guirao (1963); Bond and Stevens (1969); Marks (1974a); -ve: Wicker (1968)	
*Saturation*	Anikin and Johansson (2019); Hamilton-Fletcher et al. (2017); Sun et al. (2018); -ve: Wicker (1968)	cf. Woodworth and Schlosberg (1954, p. 364)	Anikin and Johansson (2019)	
*Shape*	Marks (1987); Parise and Spence (2012)	Adeli et al. (2014); Arai et al. (2021); Gurman et al. (in press)		
*Richness*				Mudge (1920)
*Contrast*	Wicker (1968) -ve: Evans and Treisman (2010)			

**Figure 3.**
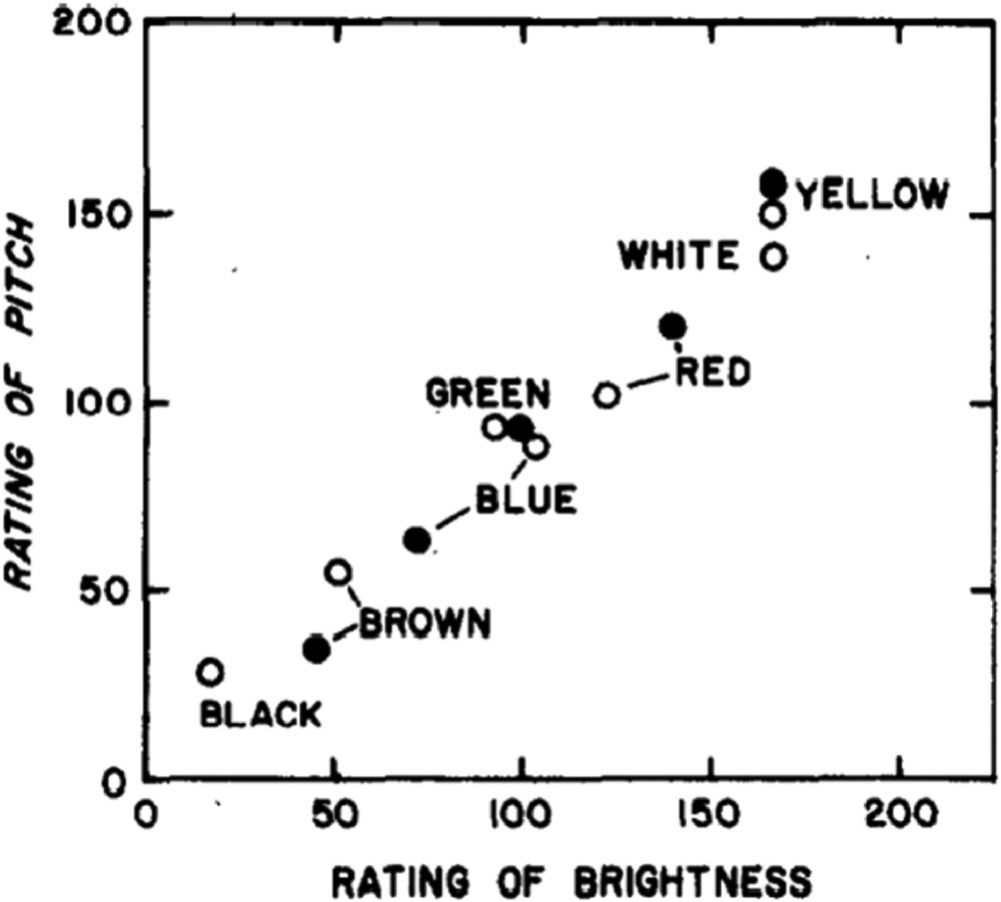
Mean ratings of pitch versus mean ratings of brightness to colour words. (Open symbols: results of Marks (1982), Experiment 2; visual scale, dark-bright. Filled symbols: results of Marks (1982), Experiment 4; visual scale, dim-bright.) [Reprinted with permission from Marks (1982, Figure 6).].

A couple of the studies that have been reported by Marks and his colleagues are interesting inasmuch as they appear to highlight the existence of a crossmodal correspondence between colour words and the associated, or corresponding, pitch in adults (Marks, 1982) (see Figure 3). A few years later, Marks et al. (1987) went on to demonstrate much the same crossmodal associations in children aged between 9 and 13 years (see Marks et al., 1987, Figure 47). Such results presumably suggest that people may associate different (prototypical) or imagined colours as having a specific brightness level, and moreover that they do so from early in development. One final point to note here concerns Simpson et al.’s (1956) suggestion that: “A third interpretation, most consistent with our finding of selective pitch-colour associations in children, is that particular hues and pure tone frequencies are associated with each other because of a certain inherent “belongingness” between each member of a given pair and a particular mood.” (Simpson et al., 1956, p. 102). As we will see later, this mood, affect, or emotion-based account of crossmodal mappings is one that many other scientists, as well as a number of artists have seemingly also stumbled across (cf. Kandinsky’s, 1977, ideas around the notion of ‘inner harmony’).

Wicker (1968) conducted a pair of studies investigating what he referred to as the “intersensory dimensions in perceptual or cognitive space, *i.e.* of dimensions which are significantly descriptive of sensory inputs from more than one modality.” [italics in original] (Wicker, 1968, p. 178). Wicker was particularly interested in “behaviour which can be conceptualized as reflecting perceptual or associative alignment between sensory attributes from different sense-modalities” (Wicker, 1968, p. 178). In a first experiment, the participants were presented with a range of 13 pure tones (300, 400, 500, 600, or 700 c.p.s.; this, note, a more sensible choice of tones than used in Simpson et al.’s, 1956, study, given that the researchers were potentially able to investigate also another variable/dimension (such as different notes) beyond higher-lower frequency…) of varying loudness (53-84 dB); they were also presented with 13 coloured Munsell colour squares (green, red, blue, and yellow) of varying brightness and saturation. The participants were instructed to rate the similarity of all pairs of tones and, in a separate experimental session, to rate the similarity of all possible pairs of colour patches. They also had to rate every individual tone and colour patch in terms of 25 semantic differential adjective scales (cf. Moller et al., 2009). They were further encouraged to rate the similarity of the auditory and visual stimuli in another part of the study. In particular, according to Wicker (1968, p. 180): “They were asked to rate the similarity of every tone to every colour on a nine-point scale.”

Multivariate scaling revealed two orthogonal alignments underlying the intersensory and cognitive space: pitch-brightness and loudness-contrast. These were established using multidimensional-scaling, semantic-differential scaling, and an intersensory transfer of training paradigm. At the same time, however, Wicker (1968) also noted how several other ‘alignments’, what in today’s parlance might well be called crossmodal correspondences (Spence, 2011), were not evidenced by his analysis of the data, namely pitch-saturation, loudness-brightness, and loudness-darkness. The failure to find the systematic relationship between pitch and hue was especially surprising given the results of Simpson et al.’s (1956) previous developmental study. Taken together, the studies reported by Simpson et al. and Wicker would therefore seem to suggest that colour-sound mapping is likely based on frequency (in terms of low-high continuum) but ignores octave repetition. In fact, if the mappings were to be based on octave repetition, then most experimental stimuli would have been matched to the same colour (as they are all octaves apart).

A few years later, Bernstein et al. (1971) conducted a small study in which four participants made speeded discrimination responses to the colour (red vs. blue) of a light which was either presented by itself, or else was accompanied by a task-irrelevant tone (of either 100 or 1,000 Hz). However, no significant effect of crossmodal mapping was observed as a function of the colour-pitch mapping.^21^ As outlined by Bernstein and colleagues, the particular motivation for undertaking their research was to test the hypothesis that: “a high frequency tone would have greater facilitatory effect for a stimulus from the high (short wavelength) end of the spectrum and a low tone would have greater facilitatory effect for a stimulus from the low (long wavelength) end of the spectrum.” (Bernstein et al., 1971, p. 1327). However, the tiny sample size means that it is unclear whether Bernstein et al.’s study was adequately powered to find an effect in the first place.

Finally in this section, Melara’s (1989) studies on dimensional interactions between colour and pitch should also be mentioned. As a cognitive psychologist, Melara was particularly interested in studying dimensional interactions between colour and pitch using the well-established Garner interference paradigm. Indeed, there had been much research interest in the preceding decades concerning Garner’s notion that there was a meaningful distinction between separable and integral stimulus dimensions. The participants in Melara’s studies had to make speeded classification responses to either black and white stimuli or to triangle waveform tones having a fundamental frequency of either 1046.5 Hz (high) or 174.6 Hz (low). That is, the participants made speeded classification responses to a sequence of stimuli presented in one sensory modality while attempting to ignore the task-irrelevant stimuli that were sometimes presented in the other modality. The results of a series of experiments suggested that the crossmodal connection between these achromatic colours (what might be described as brightness) and pitch was partly strategic and partly mandatory (cf. Sun et al., 2018). It is, though, important to bear in mind here that evidencing congruency effects in speeded classification tasks such as these does not, in-and-of-itself, necessarily tell us anything about the perceptual similarity of the stimuli that were used. That is, congruency effects in behavioural studies have multiple causes, only a minority of which are likely to be perceptual in origin.

**Figure 4.**
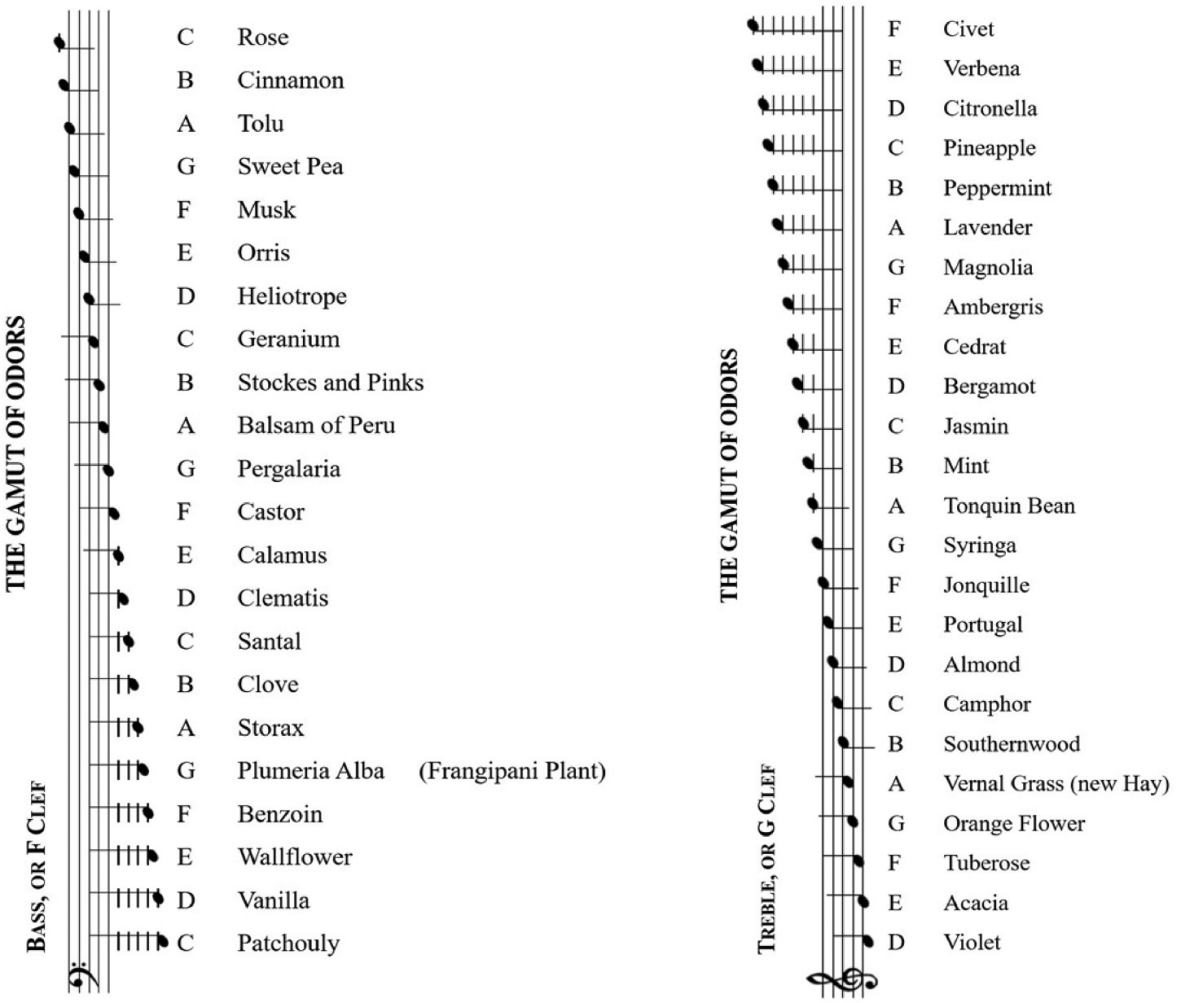
Scale of crossmodal correspondences between sound and odours reproduced from Piesse (1867, pp. 42–43).

One other point to note here, in passing, is the very different pitch ranges that have been used in the studies reported above. That is, while the label ‘high-pitched’ might be associated with a pure tone of 1,000Hz in one study (e.g., Bernstein et al., 1971) the same label has been associated with a 12,000Hz tone in another (Simpson et al., 1956). The assumption amongst many of the experimental psychologists would therefore implicitly seem to have been it is the relative position of stimuli along their respective continua that matters (cf. Spence, 2019, for a review). But, one might ask, is that necessarily the case?^22^ What is also worth noting is that most (if not all) of the above-mentioned studies used pure tones, which are not properly musical sounds, as they lack overtones (and therefore timbre). Since fundamental aspects of frequency perception (e.g., discrimination) are shown to be facilitated (in healthy participants) with complex or harmonic tones rather than pure tones (e.g., Novitski et al., 2004; Zeitlin, 1964), this would make the choice of pure tones even more problematic, for those wanting to focus on audiovisual correspondences based specifically on frequency. It is relevant here to note that with his ‘Gamut of Odors’ (see Figure 4), Septimus Piesse has also been taken to have highlighted a direct one-to-one correspondence between musical notes and scents. Relative correspondences presumably suggest a belief in some kind of structural analogy rather than a direct perceptual mapping based on the phenomenological similarity of the component stimuli.

### 3.2. Interim Summary

Taken together, the experimental research that has been conducted to date to test the psychological correspondence between colour (hue) and pitch has failed to reveal any convincing evidence of such a mapping (or alignment of dimensions), at least not when brightness and saturation have been carefully controlled (cf. Dailey et al., 1997; Hubbard, 1996). Given these null results, researchers would subsequently appear to have been dissuaded from further searching for hue-pitch correspondences. Indeed, as Kubovy and Van Valkenburg (2001, p. 115) put it: “As profound differences between light and sound became clear in the twentieth century, psychologists abandoned the explorations of parallels between pitch and colour.” Instead, it would appear from the psychophysical research reviewed here (see Wicker, 1968) that the more natural correspondence may instead actually be between pitch and brightness (cf. Bleuler & Lehmann, 1881; Flournoy, 1893; Marks, 1982) and between loudness and contrast.^23^

### 3.3. Pitch-Brightness Correspondences

Crossmodal correspondences between auditory pitch and visual brightness/lightness have often been reported in the psychology literature (Marks, 1987; see also Sabaneev & Pring, 1929; see Table 4). What is more, according to Marks (1978, 2011; Marks & Bornstein, 1987), a neurophysiological explanation for this particular mapping may be based on the existence of common, underlying sensorineural codes (of intensity, brightness, duration, etc.). So, for example, Marks points to the fact that the auditory system appears to use temporal patterning (neural response frequency) in the coding of both pitch and loudness,^24^ and believes that this may help to explain the commonalities between both of these dimensions and visual brightness (e.g., see Marks, 1991, p. 193). This suggestion, which echoes an earlier one made by Stevens (1957) regarding the common coding of increased intensity in different sensory modalities in terms of increased neural firing, can be seen as an account of at least certain correspondences motivated on the basis of a similarity in the neural processing/encoding principles (cf. Ellermeier et al., 2021; Walsh, 2003). Spence (2011) labelled those correspondences that are putatively based on such neural similarities as ‘structural correspondences’.^25^ Nevertheless, despite the absence of robust empirical evidence supporting the existence of pitch-hue correspondences, a number of other crossmodal correspondences between dimensions of colour and sound have been documented over the last decade or so, including between timbre and hue (e.g., Adeli et al., 2014). For instance, Hamilton-Fletcher et al. (2017) examined how sound influences chroma or hue when properly controlling for lightness. To address this question, they had their participants physically adjust equiluminant colours until they matched with certain sounds. They found that, for pure tones, increases in frequency were associated with increases in chroma. Increasing the loudness of pure tones also increased chroma. Intriguingly, for complex sounds that share the same bandwidth of frequencies (100-3,200 Hz), but that vary in terms of which frequencies have the most power, all of the stimuli were associated with yellow hues. Based on their results, the authors suggest that the presence of frequencies higher than a certain threshold (i.e., above 800 Hz) consistently yields yellow hues.

**Table 4.** Summary of the various different types of crossmodal correspondence that have been proposed that have to connect auditory and visual stimuli and selected literature sources suggested and/or supported them.

Type of correspondence	Description/ Explanation	Supporting references
*Structural*	Correspondence based on structural alignment of stimulus dimensions (e.g., both pitch & hue being circular dimensions)	Newton (1704); Rimington (1895); Gombrich (1960); Garner (1978); Wells (1980); Sebba (1991); Pridmore (1992)
*Physical*	Correspondence based on fact that stimuli themseves are of a similar type (e.g., both sound & light being considered as waves)	Schellen (1872); Caivano (1994); & see Kubovy and Van Valkenburg (2001)
Physiological^*^	Correspondence based on similar neural hypothetical similar neural encoding principle (e.g., increases in intensity being represented by increased neural firing)	Stevens (1957); Marks (1978, 2011); Marks and Bornstein (1987); Marks (2011)
*Psychological* -Perceptual (-Amodal)	Correspondence based on perceptual similarity between the component stimuli (sometimes referred to as intersensory equivalence)	Kandinsky (1977); Von Hornbostel (1931, 1950); Marks et al. (1987); Smith (1987)
-Mood-based/ Affective / Emotional-mediation	Correspondence based on mood/emotion associated with each of the component stimuli being similar	Sabaneyev (1911), as cited in Galeyev and Vanechkina (2001); Mudge (1920); Simpson et al. (1956); Cutietta and Haggerty (1987); see Spence (2020a) for a review
-Associative/ Statistical	Correspondence based on associative learning / internalization of crossmodal statistical regularities in environment	Critchley (cited in Harrison, 2001); see Spence (2011) for a review

* Note that Spence (2011) originally labelled this category ‘Structural'. However, given that this label had already been introduced 20 years earlier by Sebba (1991) to describe dimensional alignment of perceptual space, it would seem more appropriate, in hindsight, to call this category 'Physiological', instead given that that is the putative cause/source of the correspondence.

Anikin and Johansson (2019) reported on a series of 22 experiments in which they investigated crossmodal correspondences between visual (luminance, hue [R-G, B-Y], saturation) and acoustic dimensions (loudness, pitch, amongst various others). Their results revealed that loudness is associated with saturation, while pitch is associated with both luminance and saturation.

### 3.4. Timbre-Colour Correspondences

As well as investigating any pitch-hue correspondences, experimental psychologists have, over the years, also assessed the nature and consistency of any timbre-colour (hue) crossmodal correspondences. In the latter case, though, the motivation behind researchers’ attempts to demonstrate such correspondences would appear to have been driven more by, or at least related to, the particularities of the idiosyncratic reports of coloured music synaesthetes rather than anything else (see Donnell-Kotrozo, 1978). For example, the composer Raff reported that he perceived the colour of the sound of the trumpet to be scarlet (other people apparently report it to be bright red; Ortmann, 1933); for Kandinsky, meanwhile, the sound of the tuba was also red. Or take Ginsberg’s (1923, p. 589) suggestion that: “Most, if not all of us, seem to agree with the following descriptive phrases; the silvery tone of the violin, the red blare of the cornet, the golden voice of the tenor, etc.” In 1899, the French musicologist, Albert Lavignac suggested a number of instrument-colour relationships such as Flute—azure blue; Oboe—green; Clarinet—red-brown; Horn—yellow; English horn—violet; Trumpet—crimson with orange; Trombone—crimson with orange; Cornet—red; Bassoon—dark brown; Timpani—black; Side drum—grey; Triangle—silver; and Violin—blue (see Donnell-Kotrozo, 1978; Lavignac, 1899).^26^

In an early study, entitled ‘The common synaesthesia of music’, Mudge (1920) assessed visual responses to music in a group of 50 mature students. The latter were instructed to report the colours or brightnesses that they associated with certain tones, keys, instruments and familiar musical compositions. While Mudge notes that there was little uniformity as to the particular colour associations (and that eight of his participants reported a total lack of such colour associations), Mudge does admit that some tentative generalizations appear to be warranted. In particular, 34 of the students thought that low tones yielded dark colours, 26 that medium tones yield medium bright colours, and 36 that high tones yielded bright colours. In terms of timbre-colour correspondences, the sound of the violin was typically reported as blue or violet (or related colours), the trombone was reported to be dark, often brown (though for some it was yellow), and the clarinet and flute were reported to be bright. Mudge concludes with the suggestion that there appears to be an association between the complexity of tones, or timbre, and the ‘richness’ of the associated colour.

In recent years, a number of other studies have been published in which significantly non-random associations have been documented between timbre and colour/hue in non-synaesthetic individuals (cf. Adeli et al., 2014) (see Table 4). For instance, Reuter et al. (2018) claim (albeit only in a conference paper thus far) to have demonstrated consistent colour-timbre mappings in non-synesthetic individuals. Meanwhile, in terms of auditory timbre-colour/shape crossmodal correspondences, Adeli and colleagues concluded that their participants: “strongly associated soft timbres with blue, green or light grey rounded shapes, harsh timbres with red, yellow or dark grey sharp angular shapes and timbres having elements of softness and harshness together with a mixture of the two previous shapes.” (see also Arai et al., 2021; Gurman, McCormick, & Klein, in press, on timbre-visual shape correspondences). It should, though, be noted that in much of the modern research, colour and visual brightness are conceptualized as a couple amongst many of the crossmodal correspondences that people may hold with specific timbres, including with basic tastes, olfactory stimuli, textures, volumes etc. (Crisinel & Spence, 2010, 2012; see also Giannos et al., 2021; Saitis et al., 2020).^27^ That is, hue-brightness to timbre crossmodal correspondences would not seem to be granted any kind of preferential status relative to the others. This state of affairs obviously contrasts with the disproportionate amount of theorizing that has seemingly been given over to the putative crossmodal correspondences between pitch and hue.

## 4. On the Artistic Exploitation of the Crossmodal Correspondence Between Colour and Sound: Colour Music and Colour Organs

While one important element, or strand, of artistic interest in the relation between colour and music relates to analogies between colour and sound, some artists and inventors have gone further in wanting to postulate an exact physical correspondence between light and sound.^28^ Indeed, the promise of there being a robust code that would help to translate visual experience into sound, or vice versa, has long been the dream of many of those artists interested in trying to create ‘colour music’ (Moritz, 1997). According to Moritz, the idea that colour and music were somehow connected fascinated Renaissance artists such as Leonardo da Vinci (who produced elaborate spectacles for court festivals), Athanasius Kircher, the popularizer of the “Laterna Magica” projection apparatus (cf. Castel, 1725, 1735, 1740; Jewanski, 2010), and Arcimboldo, who produced entertainments for the Holy Roman Emperors in Prague. In the Sixteenth Century, the latter artist also conceived of a colour music (Eastlake, 1840; cf. Spence & Di Stefano, 2022). Meanwhile, Erasmus Darwin (1790) proposed a luminous music, to be played with coloured lights that were to be synchronized to the sounds of a harpsicord. Intriguingly, both Castel and Darwin believed that there was a certain ‘natural’ relation between colours and sounds. For Castel, whose colour organ was first exhibited in 1735, each note of the scale corresponded to a specific colour. In particular, the Jesuit priest assigned blue to *do*, green to *re*, yellow to *mi*, and red to *sol,* not arbitrarily or on whim, but because of some believed intrinsic appropriateness (see Marks, 1975, p. 313; see Table 2).

Colour music can be seen as just an extreme manifestation of the concept of musical analogy in the visual arts (Zilczer, 1987, p. 101), and while a number of artists were happy to merely title their paintings with musical terms, others have wanted to go further (cf. Vergo, 2012; Zilczer, 2016). As Zilczer (1987, p. 102) notes: “Since the late nineteenth century, musical analogy in the fine arts has taken a variety of forms which range from simple parallels to more complex systems of correspondence between the visual and musical arts.” Zilczer (1987, p. 101) suggests that the term ‘colour music’ was coined in the closing decades of the 19^th^ Century to describe a visionary new art form, created by means of coloured lights and independent of easel painting. As the colour theorist Maud Miles put it: “Perhaps some genius will invent a pipe organ behind a screen of coloured lights. If these same lights could be operated by the same keys that play the organ, and if they could be reduced in brilliancy as the music grows softer, then a nearly perfect music and colour parallel would be produced.” (Miles, 1914, p. 97).

The synaesthetic Russian composers Rimsky-Korsakov and Scriabin both came out with their own distinctive colour-tone mappings at around the same time (see Table 5, for a comparison). That said, according to a survey by Vanechkina (1968; cited in Galeyev & Vanechkina, 2001), most Russian practitioners tended to follow Rimsky-Korsakov’s mappings rather than those of Scriabin. Galeyev and Vanechkina suggest that Scriabin built up his particular system of colour-tonal analogies, deriving many of the correspondences theoretically, rather than based on his own well-documented synaesthesia (Myers, 1914; though see Harrison, 2001). What is more, it should also be remembered here that Scriabin’s composition apparently did not match neatly with his synaesthesia. Note, once again, the theoretical rather than perceptual motivation underpinning the mapping. According to Galeyev and Vanechkina (2001, pp. 359–360): “Scriabin’s analogies are not so mechanistic in their motivation: his correlations are based on the equivalence of “complexity” of tonalities and colour (Schumann has defined tonality “complexity” as the number of alteration signs in the designation of tonalities; in its turn, colour “complexity” can be defined according to its place in a spectrum: colours at the red end are “simpler” than colours at the blue end)!”

**Table 5.** Colour-sound correspondences proposed by the Russian composers Rimsky-Korsakov and shortly thereafter by Alexander Scriabin. Note that absence of ‘major’ designation in Scriabin’s list is common in 20^th^ century music. Here it might be wondered whether Rimsky-Korsakov is referring to keys (major) whereas Scriabin is referring to isolated tones. [Adapted from the text of Galeyev and Vanechkina (2001).].

Rimsky-Korsakov	Scriabin
C major: white	C: red
G major: brownish-gold, light	G: orange-pink
D major: daylight, yellowish, royal	D: yellow
A major: clear, pink	A: green
E major: blue, sapphire, bright	E: whitish-blue
B major: gloomy, dark blue with steel shine	B: similar to E
F sharp major: greyish-green	F sharp: blue, bright
D flat major: darkish, warm	D flat: violet
A flat major: greyish-violet	A flat: purplish-violet
E flat major: dark, gloomy, grey-bluish	E flat: steel colour with metallic sheen
B flat major: darkish	B flat: similar to E flat
F major: green, clear (colour of greenery)	F: red, dark

**Figure 5.**
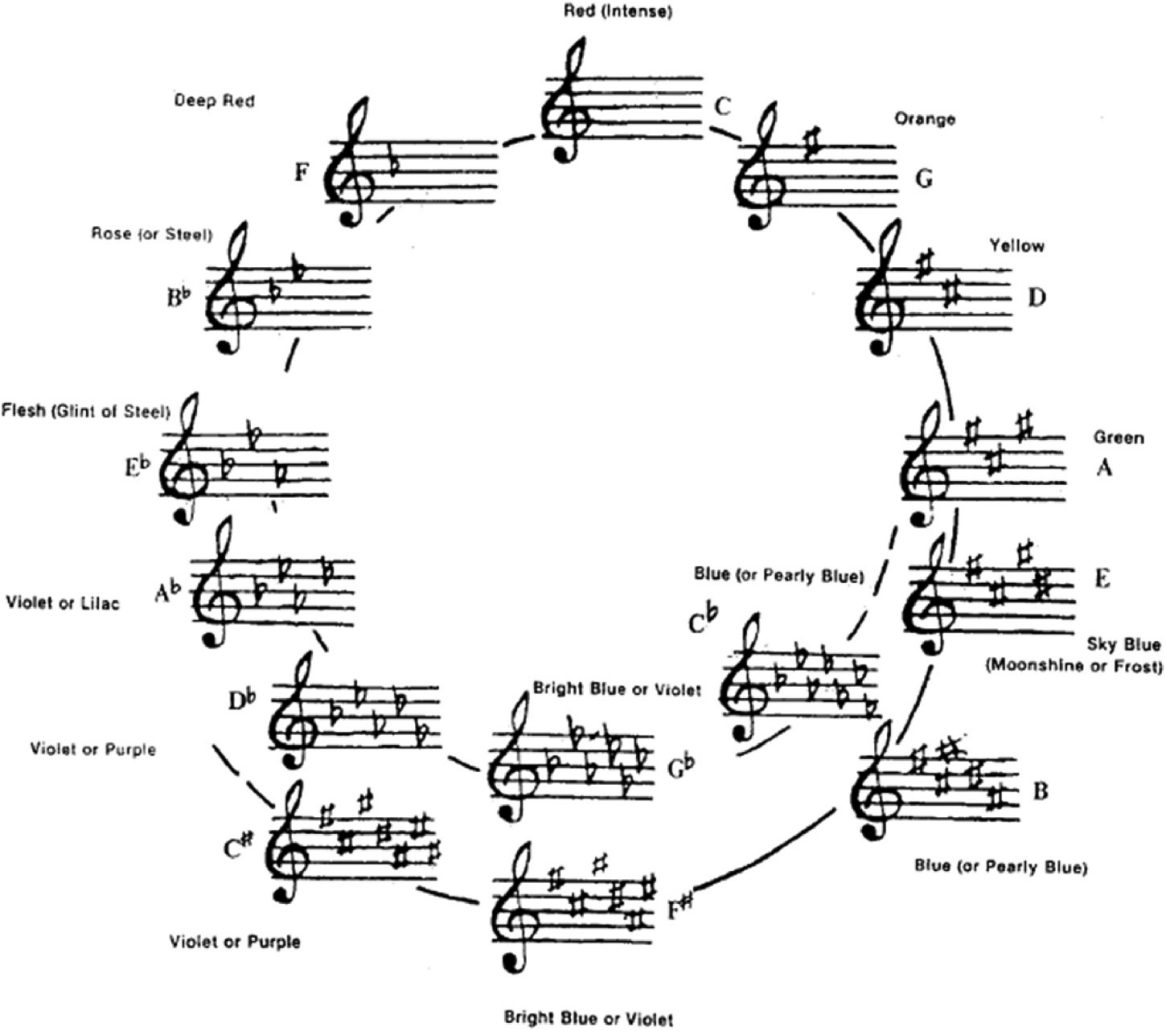
The scheme of “colour hearing” correspondences, by A. N. Scriabin. [Reprinted with permission from Galeyev and Vanechkina (2001, Figure 1).].

Complicating matters somewhat, while the experimental psychologists have tended to present pure tones (i.e., from a tone generator), in a musical context, one is much more likely to experience chords instead. For instance, just takes the colours proposed by Scriabin to accompany his Prometheus: Poem of Fire (‘light symphony’; Galeyev & Vanechkina, 2001). In this case, the colours were not correlated with individual tones in a chord structure but rather with tonalities and chordal complexes. Scriabin’s harmony in Prometheus “was practically outside the framework of the traditional major-minor system.” (Galeyev & Vanechkina, 2001, p. 359), and, as such, it would therefore be a mistake to associate the colours in the separate tones of the “octave spectrum” (see Figure 5).

Cutietta and Haggerty (1987) conducted an intriguing study on colour-music association in several hundred participants aged between 3 and 78 years. The participants were asked to listen to short excerpts (30 s) from the following three music compositions: Gustav Holst’s Suite No. 1 in Eb, third movement, “March”, Modest Mussorgsky’s Pictures at an Exhibition, fourth movement, “Bydlo”, and George Friedrich Handel’s Music for the Royal Fireworks, “Bourree”. While listening to each music example, the participants were asked to indicate the colour which the music reminded them or made them think of, or that they associated with the music (orange, yellow, green, blue, purple, red). Interestingly, the authors pointed out that no more than 5 of the 350 subjects asked for any clarification as to what was meant by a “colour association” with music. Overall, the results tend to show that when asked to associate colours with music, a large percentage of participants were homogeneous in their colour choices (e.g., barely any participant associated the Music for the Royal Firework to blue, while more than 50% aged in their 20s, 30s, 60s, 70s associated it with yellow). Consistency of colour associations seems to emerge suddenly at around 9 years of age.

Cutietta and Haggerty (1987) interpreted their findings as contrasting with the idea that colour-music associations result from the experiential conditioning. Instead, they suggest that “results can best be explained by a theory that hypothesizes that colour associations to music are the result of some sort of sensory processing of music that appears to be widespread and consistent across a wide age spectrum” (Cutietta & Haggerty, 1987, p. 89). They also suggest associations might emerge from a processing “related to emotional responses” (pp. 89-90), that might partially explain fluctuation of associations to colours during development. It is important to note the mechanisms that might drive the associations between music and colour in this study need not necessarily be similar to those that researchers have focused on when considering correspondences involving pure tones. In fact, when using extended musical excerpts, it can be hard to determine which feature(s) of the stimulus may have prompted any consensual associations that are reported (e.g., average pitch, melodic contour, timbral or rhythmic aspect). As such, any structural organization of those auditory stimuli could hardly be meaningfully applied to musical excerpts such as the ones considered by Cutietta and Haggarty (and see Spence, 2020a, for a review of the literature on colour-music correspondences).

### 4.1. Rimington’s Colour Organ

**Figure 6.**
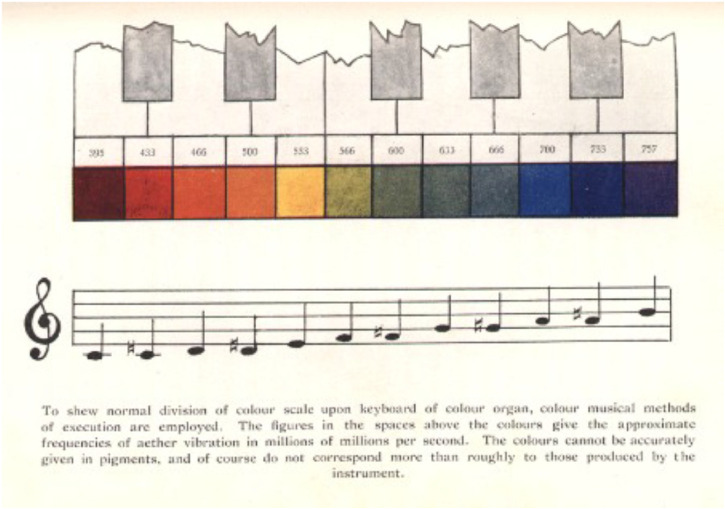
Illustration of the colour-musical note mapping that was adopted by Rimington (1895) for the first performance of his colour organ in London.

Alexander Wallace Rimington (1854–1918), a British inventor and professor of fine arts, based in London, built one of the first colour organs, patented in 1893. The first public performance of his colour organ took place in June, 1895, at St. James Hall, London (Rimington, 1895).^29^ Rimington believed in the physical equivalence of light and sound, and in his compositions, attempted to apply three musical functions, namely time (possibly referring to tempo), rhythm, and instantaneous combination (slow or rapid and varied) to colour. Zilczer (1987, pp. 118–119) notes that: “Rimington equated the seven spectrum bands of natural light with diatonic intervals which composed the musical octave.” (see Figure 6).^30^

Interestingly, however, a closer inspection of Rimington’s original address, given at the start of his first public performance in London, reveals that while the inventor stresses the physical analogies between vision and hearing, he leaves open the question of what he calls the psychic consequences. As Rimington puts it: “It will be a question of opinion, and of further experiment, whether the close physical analogy between the octaves of colour and sound has its physiological and psychical counter-part.” In fact, Rimington’s (1895) address is worth quoting at some length here, given the clarity with which he pursues the physical similarity and structural analogies between colour and music:

“Taking the spectrum band as the basis of all colour, there are two remarkable points of resemblance between it and the musical octave, which have long been commented upon and discussed. The first of them is that the different colours of the one, and the different notes of the other are both due to various rates of vibration, acting on the eye or the ear. This is very simply and clearly put by Professor Schellen in his great work upon spectrum analysis. ‘Different colours,’ he says, ‘are produced by the different degrees of rapidity with which the ether vibrations recur, just as the various notes in music depend upon the rapidity of the succession of vibrations of air.’ In a word, ‘colours are to the eye what musical tones are to the ear….

I will therefore pass to the second and equally remarkable analogy between the octave of colour and the octave of sound. If we measure the rate of vibration at the first visible point at the red end of the spectrum, we shall find it is approximately one-half what it is at the extreme violet end. Now in music, as we all know, this relationship is the same. If we take the first and last notes of an octave (by which I mean the twelfth) the latter has nearly double the number of air vibrations – and the first note of the new octave has exactly double. This, as we have seen, is the case also with the spectrum band so far as the one octave is concerned; the lowest red stands for the first note of the octave, and the highest violet for the twelfth or last note. Further than this, the blue end of the spectrum shows a tendency to a return to red in the violet, and the red end of the spectrum shows a similar tendency towards a reappearance of blue, in the fact that it passes from scarlet to carmine before it fades away, so that Sir John Herschel and others may have been right when they surmised that, if our eyes could see them, the colours of the visible spectrum would probably repeat themselves in successive octaves, in the great invisible portions beyond the red and the violet.

Starting from these remarkable physical analogies, I have divided the spectrum band into diatonic intervals or notes, on the same plan as that of the musical scale.’

It is pertinent to note that the only claim Rimington makes in support of his suggestion that his colour organ successfully translated music into visual form was the fact that apparently great pieces of music played on the colour organ give rise to pleasing effects.^31^ Here, one is reminded of similar claims concerning the scent organ (see Spence, 2021, for a review). Rimington wrote: “That colour, like sound, is capable of expressing artistic emotion there can, I think, be no question, but whether it expresses it in the same way as music is doubtful. It is, however, a somewhat strong argument in favour of the existence of the physiological and psychical analogy, that when we avail ourselves of the works of great musical composers for the interpretation of the new art, the results are vastly superior in variety, delicacy, and beauty of colour to those hitherto obtainable by other methods.” One can also find a similar appeal to the colourfulness of the transposition in Pridmore (1992, p. 60) when discussing what the Beatles’ music would look like when transduced by one of his sound-to-light convertors: “Interestingly, all-time famous pop bands, e.g., The Beatles, play very “colourful” music.” (see also Garner, 1978, for speculation on what the opening notes of “God save the Queen” would look like if rendered sequentially in colour). Again, though, one is left wondering how pleasant a control group of random sounds/music would be?

### 4.2. Other Artists Subsequently Interested in Colour-Music

The artist Max Weber (Weber, 1916), just like Kandinsky (1977), was also inspired by synaesthesia,^32^ and by the emotional correspondences that he though existed between painting and music (note here Kandinsky’s notion of ‘inner harmony’). A similar appeal to emotion can also be found in Sabaneyev (1911, p. 200; trans. in Galeyev & Vanechkina, 2001, p. 358) who writes that: “Colours, on the one hand, and sounds, on the other hand, engender various moods, often similar to one another, therefore—the associations of colours and sounds arises.”

According to Zilczer (1987), the North American architect Claude Bragdon, who was also a fan of colour music, put on a couple of colour music shows in New York (one in 1915 and the other in 1916). Bragdon based his notion of colour music on the psychological, rather than the purely physical analogy between light and sound. In his 1918 book, *Architecture and democracy*, Bragdon writes: “If we are to have colour symphonies, the best are not likely to be those based on a literal translation of some musical masterpiece into colour according to this or any theory, but those created by persons who are emotionally reactive to this medium, able to imagine in colour, and to treat it imaginatively.” (Bragdon, 1918, p. 139). In his work, Bragdon was therefore keen to enlarge the concept of colour music beyond a ‘simple’ physical correspondence between colour and music (see also Bragdon, 1916). Meanwhile, according to Zilczer (1987, p. 122), the Philadelphia pianist, May Hallock Greenewalt (active at around the same time) also promoted colour music. She apparently agreed with Brandon on the importance of the psychological basis for musical analogy, by which we might infer the common mood associations of colour and music.

Artist Stanton Macdonald-Wright, who together with Morgan Russell invented the style ‘Synchromism’ (see South, 2001; Zilczer, 1987), believed that an exact correspondence between the colour spectrum and the musical scale would provide a key to their new art. He later wrote: “For many years there has been growing a conviction that there is some deeply rooted, recondite analogy between colour and sound. Both are demonstrably vibratory; both have a varied and defined emotional stimulus for us, and each is used as a medium for an art.” (Macdonald-Wright, 1924, p. 14). This suggestion is curious inasmuch as it appeals both to an exact correspondence between colour and music while also invoking the importance of emotion.

Meanwhile, in the Soviet Union (as was), the Group 'Prometei' carried forward Scriabin’s dream of synthesizing electric light and music in the intervening decades since the composer’s death. According to one commentator: “They explored correlations between the two fields in a number of different ways: (a) correlations with individual qualities of music (pitch, key, timbre, and harmony); (b) correlations with musical themes; (c) correlations with different qualities and themes of music; (d) the polyphonic (contrapuntal) audio-visual integration approach.” (see Galeyev, 1976; Pridmore, 1992).

According to Zilczer’s (1987) excellent summary of the literature on colour music, those working in the field of contemporary art would largely seem to have lost their former fascination with the synaesthetic coloured hearing. As Moritz’s (1997) review makes clear, a number of other artists/film-makers worked with developing abstract colour displays to pair with music. One might think of Sergei Eisenstein’s (1898-1948) interest in sound-colour montage (see Harrison, 2001, p. 133). Eisenstein also wrote at one point that there is no “pervading law of absolute meanings and correspondences between colours and sound.” (quoted in Harrison, 2001, pp. 133–134). Disney’s Fantasia, with music conducted by Leopold Stokowski, can perhaps also be seen as a natural extension of the colour music movement (Pridmore, 1992), though a key strand of the emerging interest was linked to the notion of audiovisual Gestalten, which relied as much on synchrony as correspondence (see also Alves, 2005; Battey & Fischman, 2016; Corra, 1973; HaCohen, 2016; Haverkamp, 2020; Kaduri, 2016; Russet & Starr, 1988; Whitney, 1980). At the same time, however, it has also become increasingly clear that there is no straightforward structural or psychological mapping between this particular pair of senses. Indeed, Vanechkina (1973) noted almost half a century ago how almost all of those he spoke to specially emphasized the associative, metaphoric nature of “colour hearing” in music and excluded from the artistic sphere any clinical cases of colour hearing.

In the 18^th^ century, Castel confidently predicted that every home in Paris would one day have an Ocular Harpsichord for recreation purposes (Moritz, 1997). Meanwhile, according to Marks (1975, p. 313): “By the late nineteenth century and early twentieth century, interest in colour organs had blossomed into what might almost be called an epidemic (e.g., Sullivan, 1914).” Nevertheless, the great enthusiasm that colour music once aroused rapidly faded, presumably because no agreed perceptual match exists – just consider the wide variety of potential mappings that have been put forward over the years.

Synaesthesia was once a popular source of inspiration for those interested in colour music. However, other accounts based on natural, physical relations (the latter based on the physical properties of the stimuli themselves and the idiosyncratic nature of the perceptual continua), or analogous mapping in Rimington’s matching on the basis of complexity have also, on occasion been proposed. And yet it is noticeable that the common explanation, mentioned, or supported, by so much of the research, both scientific and artistic in origin, is the affective correspondences resulting from shared affect, mood, or emotion of the component stimuli. That said, there would appear to be a growing realization of the potential relevance of crossmodal correspondences to the study of music (Eitan, 2017; Timmers, 2022; Walker, 2016), or as an additional means of illustrating the ‘meaning’ of music for music students (Donnell-Kotrozo, 1978; Wells, 1980; see Bresin, 2005, for an attempt to illustrate the expressivity in music performance by means of the use of colour to link to specific emotions; cf. Clarke & Costall, 2008; Kandinsky, 1977).

## 5. Theoretical Accounts of the Structural Similarity of Musical Tones and the Colour spectrum

A complex and convoluted debate has been raging in journals such as *Leonardo* and *Colour Research and Application* (Caivano, 1994; Davis, 1979; Garner, 1978; Pridmore, 1992; Wells, 1980) concerning theoretical justifications for the appropriateness of particular structural mappings between hue and pitch. It is noticeable how those commentators contributing to this debate have come from a wide range of disciplinary backgrounds including experimental psychology (Garner, 1978), architecture (Sebba, 1991), art (Davis, 1979), and music education (Wells, 1980; see also Kandinsky, 1977, p. 25; and O'Callaghan, 2008, for philosophical interest in the relation between sight and sound). For instance, in a brief piece published in *Leonardo*, the psychologist Wendel Garner provided a particular logical foundation for correlating sound and light frequencies according to a common octave principle. However, in response, other commentators (such as Davis, 1979, a visual artist by training) subsequently pointed to the fact that there are, in fact, numerous possible correspondences between colour and music, and hence that there is no need to limit oneself to a literal structural translation of one medium to another.

**Figure 7.**
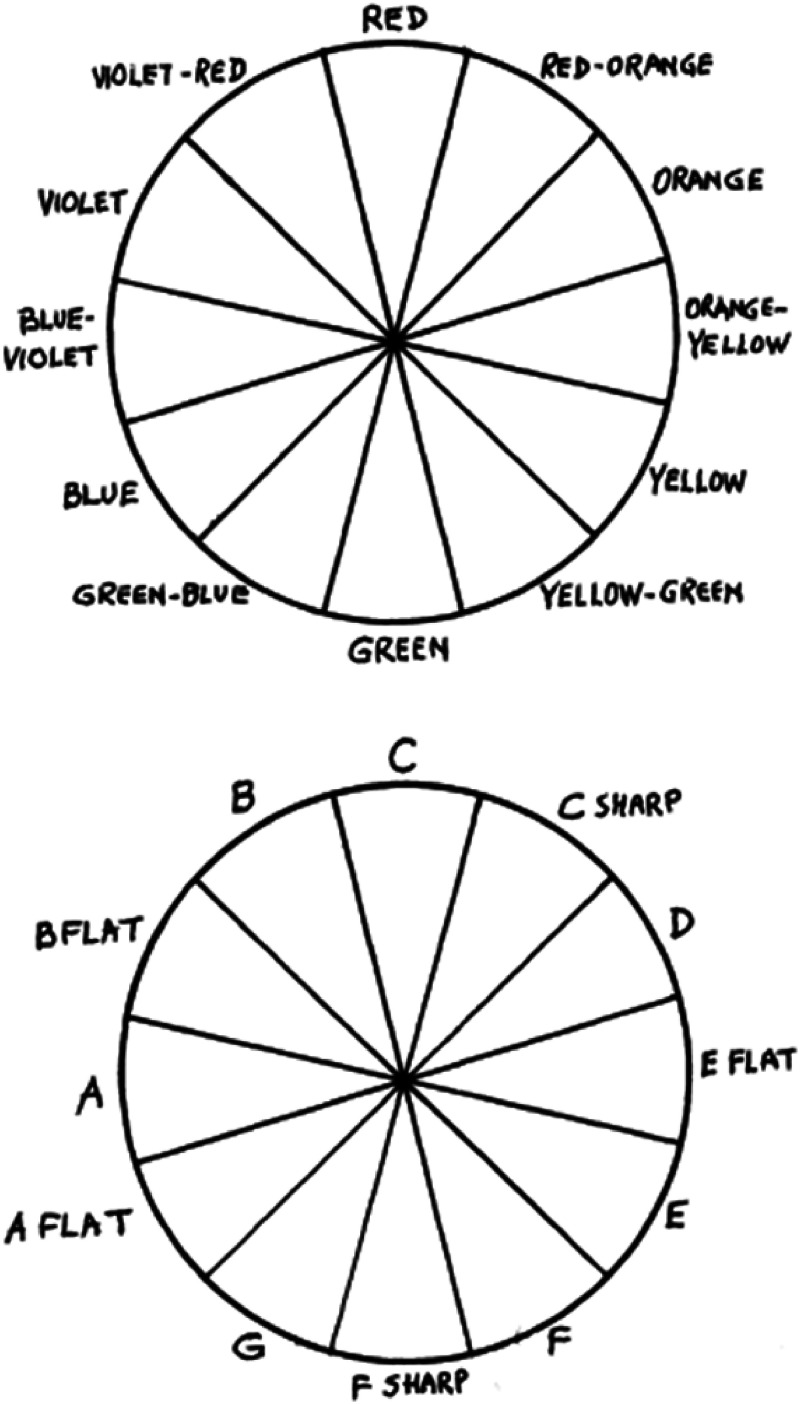
Top: colour circle. Bottom: Chromatic musical scale circle, according to Wells’ (1980) complementary account. [Reprinted from Wells (1980, Figure 1), with permission.].

As Wells (1980, p. 106) notes: “In them one would not deal with individual tones in a chord structure but would consider only the root tone as providing the chord’s colour. There is an analogy here with the harmonics of a fundamental that are not heard individually but blend together to reinforce and give character to the sound of the fundamental.” According to Wells (1980, p. 106): “I sought neither to expand nor to limit what appeared evident to me—that there was a correlation between colour and music based on the principle of complementarity.” In particular, Wells (1980, p. 101): “points out that an equal division of the musical octave into 12 half-steps permits one to recognize chords built on tones occurring at the interval of half octave or the tritone interval as being complementary to each other. This corresponds to the equal spacing of 12 hues on a colour circle in which complementary hues are located diametrically opposite each other. A circular form of the musical octave divided according to the chromatic scale of 12 half-steps places tones serving as roots for complementary chords diametrically opposite each other also.” (see Figure 7). Such a neat arrangement would not be possible with the 7-step diatonic scale that Newton was probably more familiar with.

Pridmore (1992) drew attention to the limited range of the crossmodal mappings proposed by Sebba (1991). While not wishing to criticize the appropriateness of the 13 mappings established by means of analysis of art students’ paintings made in response to specific pieces of music, Pridmore highlights the fact that there are something like 100 semitone intervals from around 30 to 15,000 Hz, what he calls the gamut of semitones (imitating Piesse’s, 1867, ‘Gamut of Odours’), while Sebba’s mappings only covered 10-13 semitones. “Tone comprises some nine cycles (octaves), each repeating the same 12 semitones, whereas hue is limited to only one cycle of hues.” (Pridmore, 1992, p. 57). Pridmore (1992, p. 57) also points to the observation that: “In psychophysical theory, any psychological correlation between music and colour must derive primarily from the physical stimuli, which, as sonic or radiant energy, have only two variables: (a) amplitude, causing loudness or brightness/lightness: and (b) wavelength, causing musical tone or hue.”

**Figure 8.**
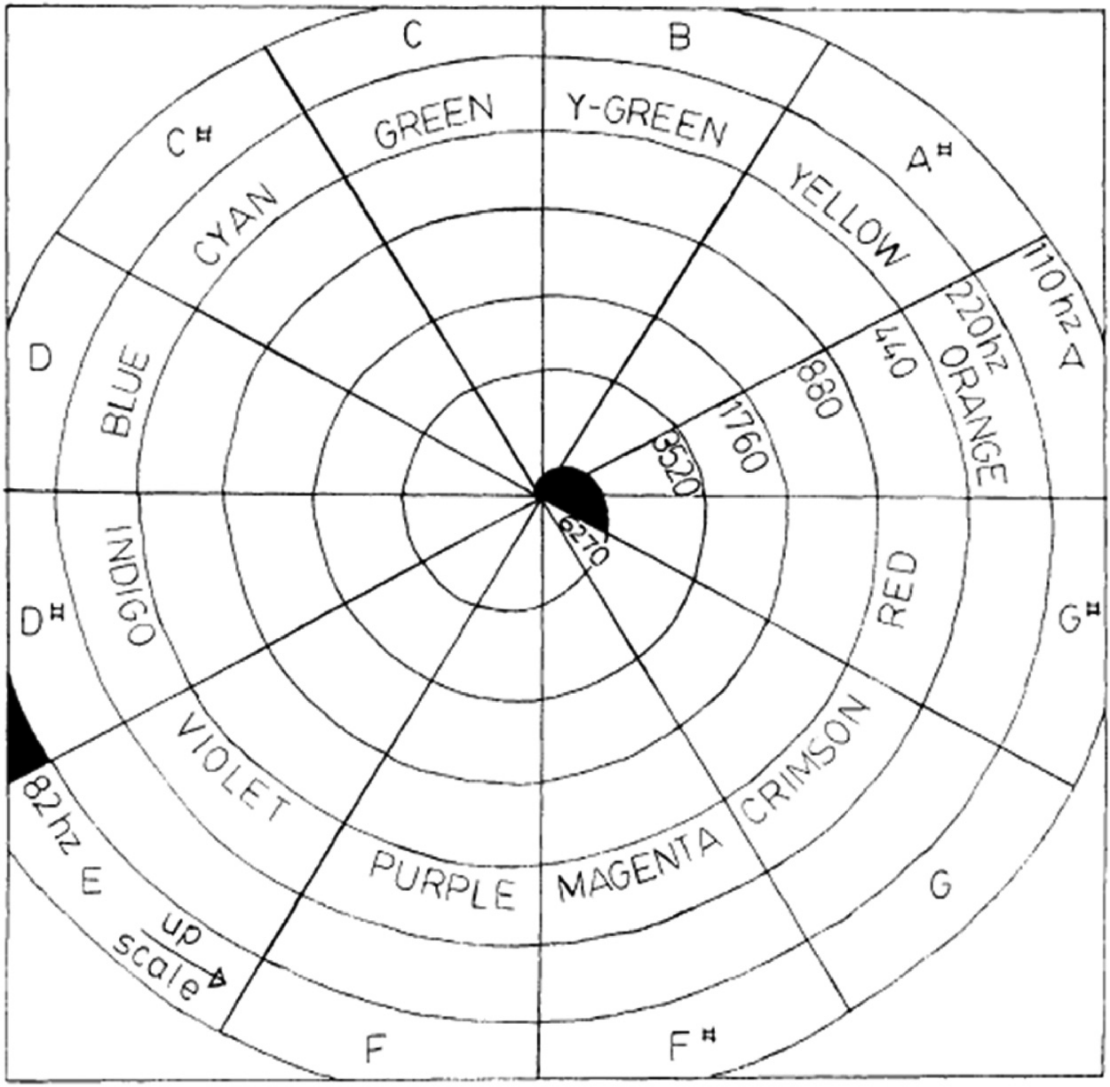
Layout of Pridmore’s (1984) final display panel. A given tone (e.g., C^#^) in all octaves is represented by a constant hue (e.g., cyan). Each octave is represented by a cycle, and each semitone (and its hue) by a sector (as presented in Pridmore, 1992).

Pridmore’s (1992) interest here is more technical, given his desire to develop a transducer capable of converting music into colour for the deaf. Indeed, he states that he intends “to support the technical or scientific approach as the only possible way of finding a constant and objective correlation (rather than arbitrary and subjective).” (Pridmore, 1992, p. 57). Pridmore (1992) describes three electronic sound-to-light transducers in order to better convey colour to give visual impression of music to students and deaf people. In the Introduction to his piece, Pridmore also writes that: “Correlation of tone and hue is also indicated by the cyclic nature of each, as octave cycle and hue cycle (or colour wheel). No other psychological dimension of sound or colour is cyclic, so no other correlation than tone and hue is possible, at least technically.” (Pridmore, 1992, p. 57). Colour mappings based on the cyclic repetition of the octave seem to reflect more adequately the fundamental acoustic property for which the octave of sounds with fundamental frequency at *f* can be expressed as 2*f*. Since octaves are labelled in the same way (e.g., C_3_, C_4_), the same tone is always associated with the same hue, e.g., C with Cyan, independent of its absolute frequency (e.g., C_3_, C_4_…; see e.g., Pridmore, 1992). Such an assumption might be the basis for experimental designs such as Simpson et al.’s (1956), mentioned earlier, in which the majority of the auditory stimuli were multiples of the same frequency. Notice here how in reference to our earlier discussion of the putative constraints on crossmodal mapping attributed to the nature of the underlying perceptual continua, we have an argument based on the nature of the underlying physical stimuli instead (see Figure 8). However, such mappings apparently do not differentiate between the same hue when it is paired to the same note at different frequencies (e.g., Cyan paired to C_3_ or C_4_).

### 5.1. Further Reflections on the Crossmodal Correspondence Between Sight and Sound

Hereinafter, we provide a list of issues regarding colour-sound correspondences that apparently limits the possibility of explaining such correspondences (or analogies; see Jewanski, 2010) by assuming any physical similarities between the two sensory stimuli.

1. The visible colour spectrum ranges between 380 nm and 740 nm (violet and red, respectively) and from 405 THz and 790 THz (red and violet, respectively). Musical sounds range from about 20 Hz to 20 kHz. Besides the huge difference in terms of the amplitude (or wavelength) of the two spectra, a relevant structural difference might rely in the non-linear distribution of tones along the frequency spectrum, in which the distance between two successive steps varies with frequency. For example, the interval C-D is about 4 Hz at 30 Hz increasing (more than 100 times) up to 500 Hz at about 4000 Hz. By contrast, in the colour spectrum, the distance (expressed in THz) between two successive hues remains constant between 30 and 80 THz (e.g., red-orange vs orange-yellow, about 30 THz; yellow-green vs green-blue, about 60 THz) (see Caivano, 1994, Figure 2).
2. Once a tuning system is chosen (e.g., Pythagorean, equal temperament…), musical tones correspond exactly to only one specific frequency in Hz. By contrast, in the case of colours, for example, 405 THz is (perceived as) red, but so too are 410 THz and 420 THz. And if one is exposed to the above colour’s patches at the same time, s/he will likely recognize three different hues of the same colour, namely red. Therefore, the sound continuum is fragmented into discrete points (i.e., tones), while the continuum of colour is fragmented into regions that might also overlap with one another (i.e., hues). One might object that a tone at 445 Hz is perceived as close to A (440 Hz), and this is indeed likely to be the case. Nevertheless, the point here is that the note is no longer A in terms of its frequency, while the hue seen at 405, 410, 420 THz still counts as red.
3. Octave periodicity, i.e., the fact that pitches with f, 2f, 3f, 4f…, are perceived as the same tone at different frequencies, is a fundamental property of auditory perception. No similar property seems to characterize colour perception. Thus, systems such as those proposed by Pridmore (1992) that conceived the colour wheel as simply repeating itself at every “octave” (this, also a feature of various online tools, e.g., see: https://www.flutopedia.com/sound_color.htm) ignore octave cyclicity (that is not mere repetition) and therefore would not be based on the structural/physical properties of the two spectra.
4. As discussed earlier, the mapping based on the correspondence between pitch and hue could not reflect structural similarities concerning the way in which stimuli are perceived in the two modalities. Other mappings based on pitch, such as pitch-saturation, as well as mappings based on other properties of sounds, such as loudness and timbre have been less investigated in the empirical literature (e.g., see Anikin & Johansson, 2019; Hamilton-Fletcher et al., 2017; and see Table 3, for a summary). However, when pitch is kept constant, differences in loudness are consistently associated to differences in luminosity (see Caivano, 1994). For example, Stevens and Guirao (1963) had their participants to use the length of a line as a variable to represent loudness and luminosity. Their results revealed that greater line length was used to indicate both greater loudness and luminosity. Other associations might be based on size of the colour patch and duration of sounds, but these seem to reflect more the similarities with respect to the temporal and spatial magnitude of sounds and colours, respectively (what some might be tempted to call amodal).

Based on the above considerations, it seems hard to explain colour-sound correspondences based on structural similarities between the component stimuli (cf. Helmholtz, 1867). Thus, it seems that two further possibilities remain. One is that stimuli are matched based on their relative position along the continuum in each sensory modality. Such an explanation (would) fit(s) with results from studies that used isolated tones, which listeners can perceive as higher or lower (such dimension might parallel, as some suggested, brightness for colours). However, when considering complex musical stimuli (e.g., Cutietta & Haggerty, 1987; see Spence, 2020a, for a review), pitch is a much more blurred perceptual correlate of sound. In such cases, probably, one needs to put forward an alternative explanation, according to which the mapping can be conceived of in terms of emotional similarities (or mediation), or even ‘inner harmony’, to use Kandinsky’s (1977) preferred terminology. Although such explanation leaves open the question about the nature of such emotional/inner harmonies, it suggests avoiding simplistic conception of mapping based on elementary properties of visual and auditory stimuli, such as brightness and pitch that seem unable to explain consistent mapping, if any.

While the popularity of colour music has undoubtedly declined, since its peak in the years around 1900, the last decade or so has seen something of a resurgence of interest in the topic of colour-sound correspondences amongst those researchers interested in the development of more intuitive sensory substitution systems (cf. Hamilton-Fletcher et al., 2016; Marks, 1983; Pridmore, 1992). In one recent study, for instance, Cho et al. (2020) investigated whether sound can be used to code colour in order to improve artwork appreciation by those individuals with visual impairments (see also Cavazos Quero et al., 2021). The question immediately crops up though as to whether certain dimension(s) of sound should be used to convey the different specific salient properties of visual images, such as colours. Is the choice an arbitrary one? Or can such systems be made more intuitive? Interestingly, in their recent research, Cavazos Quero and colleagues chose to combine sound with scent, thus presumably adding to the complexity of the process of sensory translation.

## 6. Conclusions

As hinted at by the findings of experimental psychologists, it is by no means clear that any structural similarity between the organization of colour and pitch is relevant to promoting either perceptual or affective similarity. At best, it might offer the basis for a more cognitive alignment of analogous dimensions (Kandinsky, 1977). Think of this as a reflection on one of the directions in which research moved, that is to the mood associated with more complex (albeit short) musical expressions (Bresin, 2005; Karwoski & Odbert, 1938), complex correspondences between music and painting (see Spence, 2020a, for a review).

At the same time, however, it should also be acknowledged that there may be crossmodal correspondences between visual and auditory arts based on cross-media artistic (e.g., historical) styles (Actis-Grasso et al., 2017; Duthie, 2013; Duthie & Duthie, 2015; Hasenfus et al., 1983). Ultimately, however, it would appear clear that no matter whether one is considering the basic correspondence between colour and pitch, the complex crossmodal correspondence between music selections and visual art works (see also Adams, 1995; Albertazzi et al., 2015; Albertazzi et al., 2020), or some combination of the two (e.g., colours to match music compositions, or less frequently-mentioned, musical note to match a painting) the emotion associated with the auditory and visual stimuli would appear to be the unifying link. Acknowledging the importance of such affective correspondences leaves open the question of whether there is any directly perceptible similarity, as Marks (2011) has claimed.

### 6.1. The Emotional Mediation Account(s)

In conclusion, what started out as intuition based on analogous mappings led to a search for exact natural, physical mappings between colour and musical notes. However, the evidence revealed a visual brightness-pitch mapping and, more recently, seemingly robust hue-timbre associations too. At the same time, however, while some have been convinced of the existence of a natural, physical, mapping perhaps based on perceived similarity between matching stimuli in the respective perceptual continua, it is striking how many of those working in this area over recent centuries have ended-up arguing for emotional mediation, sometimes referred to in terms of affect, mood, or what Kandinsky (1977) referred to as ‘inner harmony’, instead. In this regard, the crossmodal correspondence between simple stimuli bears close relation to the search for crossmodal correspondences amongst more complex visual stimuli, such as paintings and pieces of music, documented elsewhere (see Spence, 2020a, for a review).

Indeed, in recent years, there has undoubtedly been growing interest amongst psychologists and practitioners in emotional correspondences between music and colour (or paintings), or emotionally-mediated (or affective) correspondences, as they are sometimes known (Spence, 2020a). The interest in the emotional mediation of colour-to-music mappings is undoubtedly one that provides one additional means of translating between sensory impressions (though perhaps is not as rigorous and objective as some commentators would have liked). Looking back, it is interesting to note how Simpson et al. (1956, p. 100) already hinted at the emotional mediation account as an “indirect” association: “And lastly, before direct associations between hue and pitch could be postulated, it would first be necessary to eliminate the possibility of indirect associations based upon parallel relationships to a common mediating variable such as mood.” The primacy of the emotional mediation account also fits with Werner’s (1934) view of perceptual experiences in infancy and early childhood as largely syncretic – that is, functionally undifferentiated and as physiognomic – that is, imbued with expression and affective properties.

And, in terms of the question with which we started, why should it be that the musical sound pitch/timbre-hue/brightness mappings have attracted so much interest over the centuries, given the many other crossmodal correspondences that are now known to exist (see Spence, 2011), it would seem that there have been multiple different drivers helping to sustain the interest in the topic at different points in time. This interest can be seen as originating from the possibility of structural analogies, from the possibility of perceptual analogies, as well as with artistic interest, technological developments (colour organs previously, and sensory substitution devices more recently), and the florid concurrents reported by coloured-music synaesthetes. The fact that we are all such visually-dominant creatures (Hutmacher, 2019), and that colour appears to play a particularly important part in driving human behaviour (Elliott et al., 2015), may also be relevant here. Ultimately, though, the fact that it is affect, mood, or emotion that appears to explain the particular affinity people experience between musical sounds and colours, no matter whether the stimuli are simple or complex (e.g., paintings and musical composition; see Spence, 2020a), means that pitch/timbre-hue correspondences are, in no way, intrinsically special.

To conclude, it is perhaps worth returning to the opening sentences of the article written by Sabaneev and Pring (1929, p. 266) almost a century ago to consider how far (or not) we have come:

“THERE was a time when it would have been regarded as absurd, or at all events as a symptom of decadence, to raise the question of a correspondence between sound and colour. And yet amongst musicians this is by no means a new problem. We know that a very long time ago there were persons, very often musicians, to whom sounds presented themselves as coloured, so to speak. Resonance evoked a colour association, and this was not a fortuitous happening but was repeated with the invariability of a law. Later on, of course, this phenomenon or, if you prefer it, this faculty, attracted a certain amount of attention amongst scholars, and the name of synopsy or colour-ear was bestowed upon it. So far it appears to be an open question as to whether this connection is organic, conditioned by certain causes of a physiological character, certain proximities or contacts of the optic and auditory nervous ramifications; or whether it is merely associative, a kind of conditional reflex, and therefore may vary with different persons, maintaining nevertheless within the given individual a certain stability.”

## Notes

1. Music pedagogists have also exploited the association between tones and colours in a series of color-coded teaching tools for music training (e.g., Kuo & Chuang, 2013; see also Donnell-Kotrozo, 1978). However, it should be noted that the empirical findings have failed to show clear advantages for such teaching methods with respect to traditional ones (e.g., see Rogers, 1991).

2. Though it should be noted that there are also occasional prothetic perceptual dimensions, such as visual lightness which, at least according to Marks (1987), differ in terms of which end of the scale people choose to associate with ‘more’ and which with ‘less’.

3. According to Mellers and Birnbaum (1982, p. 593): “Two prominent theories of cross-modality matching are *mapping* theory and *relation* theory (Krantz, 1972; Shepard, 1978). According to mapping theory, psychological values of stimuli from different continua are mapped onto a common scale of sensation and can be compared directly. A cross-modality match is presumed to occur when equal strength sensations are elicited by stimuli on different continua. According to relation theory, relationships (e.g., ratios) between pairs of stimuli from different continua are compared. In physical measurement, a mass in grams cannot be compared with a length in centimeters but *ratios* of masses can be compared with ratios of length. By analogy, it may be possible to compare the ratio of the heaviness of two weights to the ratio of the loudness of two tones, since the ratios of stimulus pairs are on a common scale. Neither mapping theory nor relation theory gives an explicit account of contextual effects due to the stimulus distribution.”

4. This might be what Wells (1980, p. 103) was getting at when he wrote that: “An equivalence of aural harmony and visual is so strongly implied in Scriabin’s later works that for me they seem to emanate from a single source.”

5. Though in her commentary Smith (1987, p. 94) writes that: “The second kind of cross-modal equivalence—and the one that concerns us here—is about similarities or correspondences between dimensions of experience.”

6. Note, here, that the crossmodal correspondences between pitch and brightness, and between loudness and brightness, are also typically present in the colour concurrents evoked by auditory inducers in synaesthetes (Marks, 1975, 1991).

7. As Marks et al. (1987, p. 34) note in a footnote: “As a general rule, psychophysicists who study cross-modal matching have concerned themselves primarily with determining precise quantitative measures of intersensory equivalence; their purpose is usually to test theoretical predictions made from psychophysical functions (which relate judgments of sensory magnitudes to physical intensities) derived for individual continua like loudness and brightness.”

8. That being said, to the extent that researchers have managed to establish meaningful crossmodal correspondences between auditory pitch and distinct olfactory stimuli (see Piesse, 1867, 1891, and his famous ‘Gamut of Odours’; see Di Stefano, Murari, & Spence, 2022, and Spence, 2021, for reviews), one might imagine that there ought to be at least some level of organization, even if it is not one that is directly perceptible to us as observers. The question is perhaps then one of if we can match olfactory stimuli with different elevations, why is it that we don’t spontaneously arrange olfactory stimuli along this particular prothetic dimension?

9. The latter comparisons complicated somewhat by the fact that olfactory stimuli are often linked to their source objects, meaning that any colour-odour match may well end up being based on semantic knowledge concerning the source object rather than a crossmodal correspondence between the perceptual or affective associations of the component stimuli (see Spence, 2020c, for a review).

10. Given the above considerations, we might therefore want to return to the question of why it is that so many commentators have searched for meaningful correspondences between this particular pair of sensory modalities, or rather specific stimulus dimensions, namely pitch and hue. The explanation for any correspondence, or so it is argued here, is unlikely to lie in the structural properties of the underlying perceptual continua.

11. Karwoski, Odbert, and Osgood (1942) distinguished between what they call ‘true synaesthesia’ and crossmodal correspondences, or ‘synaesthetic thinking’; the latter involving the drawing of intersensory metaphors (see also Odbert, Karwoski, & Eckerson, 1942; and see Deroy & Spence, 2013, for an updated argument in support of the distinction between synaesthesia and the crossmodal correspondences).

12. Harrison (2001) has even questioned whether Scriabin was actually a synaesthete at all.

13. Though, on describing the multitude of synaesthesias that he himself experienced, Ginsberg (1923) notes that the ordering of colours in response to musical notes of his own coloured music does not correspond with the ordering of colours in the visible spectrum. As he puts it: “As the tones run through a series of dark, heavy, massive bass notes to notes gradually lighter, less massive and more tinkling, so too the colors run through a series from dark, heavy colors, to lighter and lighter colors, until grey is reached.” (Ginsberg, 1923, p. 584).

14. Indeed, although beyond the scope of the present review, the literature on the colours that are associated with vowel sounds is also likely to be relevant (Cuskley, Dingemanse, Kirby, & van Leeuwen, 2019; Kim, Nam, & Kim, 2017; Miyahara, Koda, Sekiguchi, & Amemiya, 2012; Wrembel, 2009; cf. Macdermott, 1940; Marks, 1975).

15. Note here how such crossmodal associations, if they exist, might have been mediated by the musical instruments that were common during this period. At the time that these authors were writing, certain instruments were mostly used to play in certain keys depending on the material/structural properties of the instruments themselves. For example, the majority of the music that was written for baroque trumpet was in C or D (a key with 2#) (though one can find rare exceptions, e.g., trumpets in Eb). Later on, famous violin concertos by Beethoven, Brahms, and Tchaikovsky were composed in D major (though you can find others in E minor or B minor, it is hard to find one in Ab major). As such, the association between bright colours and keys with sharps can be explained through the associations between keys with sharp(s) and the trumpet and then between the trumpet and bright colours. However, the association between emotional or affective features and musical keys, as allegedly experienced by Goethe, has hardly been documented in the empirical literature. For example, Powell and Dibben (2005) demonstrated that participants were not able to identify the mood from the key or the key from mood, nor to experience a change in perceived mood of a piece if it was performed in a different key. Thus, they suggested that, at least in equal temperament, all keys are perceived as identical. On the other hand, Hailstone, Omar, Henley, Frost, Kenward, and Warren (2009) demonstrated that timbre (i.e., the musical instrument) affects the perceived of emotion conveyed by a melody, while Murari and colleagues (2020) suggested that key clarity, rather than merely key, likely plays a role in prompting colour-sound associations (see also Murari et al., 2015 for the association between minor/major mode and colours).

16. Note that the latter claim is not entirely clear, given that G-b should be a third beyond the octave.

17. According to Zilczer (1987), the results of Helmholtz’s optical research was disseminated to English-speaking artists via Arthur Herber Church’s book on colour for students, first published in 1872 (see Church, 1872; see also Koenigsberger, 1965, pp. 174-175, on Helmholtz's refutation of the analogy between colour and sound). Helmholtz wrote that “this comparison between music and colour must be abandoned” (1878/1971).

18. The choice of frequencies in this case is somewhat idiosyncratic in that pure tones at 125 (f), 250 (2f), 1000 (8f), 4000 (32f), 8000 (64f) are perceived as the same tone though several octaves higher. This choice might be reasonable if the researchers wanted to investigate only the association between frequency (higher-lower) and colours keeping the musical tone unaltered (i.e., the same note).

19. According to Simpson et al. (1956), Argelander (1927) obtained similar results in adults some years earlier.

20. Misleading in the sense that synaesthesia proper tends to have a conscious concurrent, and is typically idiosyncratic in terms of the specific colour chosen to match a given sound. Neither of which would to have been demonstrably the case here (see Deroy & Spence, 2013).

21. Bernstein et al. (1971) seem to have been unaware of Wicker’s (1968) earlier study, or at the very least chose not to cite this research.

22. Here, it is perhaps worth noting that while the literature more generally does indeed show certain crossmodal correspondences to be relative (especially those involving auditory pitch; see Spence, 2019, for a review), others, such as the pitch-elevation correspondence, appear to be modulated by the absolute pitch of the stimuli used (e.g., Parise, Knorre, & Ernst, 2014).

23. Evans and Treisman (2010) also failed to demonstrate any crossmodal correspondence between visual contrast and auditory pitch in their speeded classification studies.

24. Note that this may only be true up to a certain frequency, as for relatively high frequencies neural firing rates are slower than sound frequencies. So the firing-frequency ratio is no longer 1:1, and presumably does not increase linearly.

25. Somewhat confusingly, however, this term is used differently by Sebba (1991). Specifically, the latter author uses the term to refer to the analogous principles of perceptual organization in audition and vision. As such, a better name for this type of correspondence might be ‘physiological correspondences’ instead.

26. Of course, the link between timbre and colour is in some sense also privileged in language. After all, the German word for timbre is *klangfarbe* (‘colour of sound’; see Galeyev & Vanechkina, 2001). As Mudge (1920, p. 345) noted a little over a century ago: “it is probable that the common term “tone-color” is not a mere figure of speech based upon analogy.”

27. One is reminded here of Titchener’s (1909, 194 ff.) observation that: “Every sensory stimulus of moderate intensity arouses a wide-spread reaction. It has been shown, for instance, that the sounding of a single note upon the harmonium will not only bring about an adjustment of the organs of hearing, but will also call out visual, verbal and other associations. ….”

28. Even though Helmholtz had disproved it in the 1860’s (see Zilczer, 1987, p. 118). Though a careful reading of the literature will be needed to know quite what it is, exactly, that Helmholtz supposedly ‘disproved’.

29. Though, for earlier colour organs, see Castel (1725, 1726a, 1726b, 1751). The Jesuit Father Louis-Bertrand Castel built an Ocular Harpsichord somewhere around 1730 (Moritz, 1997).

30. Although not performed in Scriabin’s lifetime, the first performance of his light symphony took place in New York in 1915, using Rimington’s colour organ (Plummer, 1915). However, here we can be pretty sure that the colour mapping incorporated in Rimington’s organ was unlikely to have been quite as Scriabin had intended given that the latter’s exact idiosyncratic crossmodal mapping was only discovered some decades later (Spence, 2020b).

31. Though this observation regards the allegedly pleasant effect of the performance played on the colour organ. However, the key point here should be on the translation between sounds and colours. In other words, one would really want to know whether an auditory cacophony would result in unpleasant visual effects.

32. The assumption in this case seemingly being that synaesthesia was a common experience of human perception (see Zilczer, 1987). Though, as Wells (1980, p. 103) notes: “there is no evidence for synaesthesia as a general human phenomenon, that is, the subjective correlation between specific visual colors and specific musical tones.”
